# New space-time block codes from spectral norm

**DOI:** 10.1371/journal.pone.0222708

**Published:** 2019-09-26

**Authors:** Carlos A. R. Martins, Mauro Luiz Brandão, Eduardo Brandani da Silva

**Affiliations:** 1 Department of Mathematics, UTFPR, Pato Branco, PR, Brazil; 2 Department of Electrical Engeneering, State University of Maringá, Maringá, PR 87020-900, Brazil; 3 DMA - UEM, Avenida Colombo 5790 - Campus Universitário, 87020-900-Maringá-PR, Brazil; Information Technology University, PAKISTAN

## Abstract

Current research proposes a natural environment for space-time codes and a new design criterion is obtained for space-time block codes in multi-antenna communication channels. The objective of this criterion is to minimize the pairwise error probability of the maximum likelihood decoder, endowed with the matrix spectral norm. The random matrix theory is used and an approximation function for the probability density function for the largest eigenvalue of a Wishart Matrix is obtained.

## 1 Introduction

A consistent theory for communication systems was introduced by Shannon’s [1] classical work, where it was proved that, for any communication channel with capacity *C*, the data transmission below *C* may be done efficiently by using an appropriate error-correcting code, or rather, there exists a code such that the error probability may become as small as required. The results of Shannon’s work are valid for channels with only one transmitter antenna and only one receiver antenna, called *Single Input and Single Output (SISO)* channel. However, during the last decade, we have witnessed a huge demand expansion in telecommunications, and the available technologies will not be sufficient in the future. This is due to the growing need to develop reliable communication systems that allow high rates of data transmission and, consequently, to the study and development of new mathematical methods and structures that provide support for new technologies, mainly wireless communication systems.

Wireless transmissions may be impaired by a number of factors, such as great distances, objects between wireless devices, and other wireless networks, which restrict communication speed and reliability. Low capacities and high error rates of fading channels, plus the growing demand for wireless devices, motivated the development of several techniques to overcome these disadvantages. The works of Foschini-Gans [2] and Telatar [3] proved that the multiple-input and multiple-output (MIMO) communication systems attained capacity of data transmission greater than SISO systems. These results proved the superiority of MIMO systems and called the attention of researchers, originating several real applications. The foundations of space-time codes were established by Tarokh, Seshadri and Calderbank in [4] and space-time trellis codes (STTC) were introduced.

A special technique which explores spatial and time diversity of MIMO channels, called space-time block codes (STBC), was first studied by Alamouti [5], and instantly became a topic of great importance in digital communications. Tarokh, Jafarkhani and Calderbank extended Alamouti’s results in [6]. These works triggered researches on criteria to design good space-time codes for MIMO channels. After the publication of these works, the constant development of the space-time block code theory gave birth to new families of codes, that can be confronted using extensive information theory and construction parameters, such as the pairwise error probability (PEP), the code rate and the diversity order. Based on the above, several design criteria have been previously proposed to design space-time block codes for MIMO channels. The rank and determinant criterion [4] is a criterion for asymptotic SNR used to design full-rate and full-diversity STBCs. For low SNRs, the trace criterion [7], also called Euclidian-distance criterion, can be used to design STBC with low pairwise error probability.

The language of matrices to model the communication systems with multiple antennas, is the natural way [8]. Random matrices are particularly a powerful tool. The theory of random matrices emerged in the work of the mathematical statistician John Wishart in [9], but it gained great visibility in the 1950s with the contributions of the physical mathematician Eugene Paul Wigner, with publications [10], [11] and [12], on the spread of resonance of particles with heavy nucleicores in slow nuclear reactions. Further, the physical mathematician Freeman Dyson formalized the theory in [13], [14] and [15]. The theory of random matrices is used in several areas and problems, such as Riemann hypothesis, stochastic differential equations, statistic physics, chaotic systems, numerical linear algebra, neural networks, information theory, signal processing and in the study of the capacity of data transmission in MIMO channels. The deep mathematical results may be perceived in [16], [17], [18] and [19]. For applications, see [20] and [21]. The study of probability density and distribution functions of eigenvalues is one of the main problems in the random matrices theory. It was attacked by von Neumann, Birkhoff, Smale, Demmel and others. The pdf of the eigenvalues of a Wishart matrix was established in 1939, in [22]. Many researchers studied this issue, for instance, [23], [24], [25] and [26]. Estimations for the largest and smallest eigenvalues are given in [27], [28], [29] and [30]. More recently, [31] and [32] have been published. These results have many applications, and they were used to study the channel capacity of MIMO channels, for examples [21], [3], [33] and [34].

Current research proposes a new approach on STBC. We assume that the space-time block codes are elements of an appropriate normed space of matrices endowed with the spectral norm as the intrinsic norm of this space. From this norm, a new criterion for the design of STBC is proposed. The maximum likelihood decoder is endowed with this norm, and several results from the theory of Random Matrices are used to obtain this criterion. As far as we know, this is the first time that random matrix theory is used to obtain a design criterion for STBC. The usual criteria and models used, even in a very recent work such as [35], neither they assume a natural environment for the space-time block codes, nor do they use the spectral norm.

We may also remark that, since its origin, MIMO has seen deep technological advances. Initially, there was the Point-to-Point MIMO [2], [3], [36], and, subsequently, the more efficient Multi-User Mimo [37], [38], [39], [40]. The main drawback of these technologies is that they are not scalable. Current State-of-Art is the Massive MIMO [41], [42], [43], [44], [45], which is a scalable MIMO technology. There are several theoretical and practical questions about Massive MIMO. A new criterion which allows matrices that cannot be used by other criteria may be a useful tool in this new context. Further, the proposed criterion can be used in all *SNR* regimes.

The work is organized as follows. Section 2 describes the basis on MIMO and gives the usual design criteria to project STBC. Section 3 introduces the spectral norm and its main properties. We propose a natural environment where STBC inhabits. The maximum likelihood decoder is endowed with the spectral norm. Section 4 exposes the Random Matrices theory, with a focus on the cumulative distribution of eigenvalues of Wishart Matrices. In this section, we obtain an approximation function to the probability density function for the largest eigenvalue of a Wishart Matrix. This approximation will be used to provide a new design criterion to project STBC. In Section 5, we introduce the new criterion to design STBC, called *Largest Eingenvalue Criterion*, and we give a bound related to it. Section 6 presents a performance analysis between the proposed criterion and the known ones provided for several known STBC codes. We present new examples of codes and family of codes. In Section 7 we apply the new criterion for codes whose matrices are made from blocks.

## 2 System model and notations

This section fowards a short review of space-time block codes. For more details, see [46]. Matrices are represented by capital letters and vectors by bold lower cases.

Consider a constellation S⊂C. A space-time block code (STBC), or simply a code, is defined as a subset of matrices $C\in S^{n_{T}\times n_{S}}=\left\{{\left\{s_{ij}\right\}}_{n_{T}\times n_{S}}:s_{ij}\in S\right\}$, where the natural numbers *n*_*S*_ and *n*_*T*_ are the number of time slots and the number of transmit antennas, respectively. Each element of a STBC is called a word. Normally a STBC is represented with only one matrix

$$\begin{array}{c} (\begin{array}{ccc} s_{11} & \cdots & s_{1n_{S}}\\ \vdots & \ddots & \vdots \\ s_{n_{T}1} & \cdots & s_{n_{T}n_{S}} \end{array}), \end{array}$$

where each entry *s*_*ij*_ is a function of *k* symbols *x*_1_, …, *x*_*k*_ codified by the block. In this representation, the entry *s*_*ij*_ is transmitted by the antenna *i* at time *j*. The *rate* of a STBC is defined as *R* = *k*/*n*_*S*_. STBC is *full-rate* if *R* = 1.

If we transmit a codeword C∈C of a given STBC, at the receiver we will have the following matrix

$$R=\sqrt{E_{s}}HC+N,$$

where *E*_*s*_ is the average power by signal in each transmit antenna, and the entries of matrix *N* are complex additive white Gaussian noises with zero mean and variance *N*_0_/2 per real dimension. The matrix $H={\left\{h_{ij}\right\}}_{n_{R}\times n_{T}}$, where *n*_*R*_ is the number of receiver antennas, is known as the *channel matrix*. The entry *h*_*ij*_ of *H* is the fading coefficient between the transmit antenna *j* and the receiver antenna *i*. We assume the Rayleigh model, where *h*_*ij*_ has normal distribution with zero mean and variance 1/2 per real dimension.

Suppose that the codeword *C* was transmitted. The procedure of maximum likelihood decoding is to choose $\hat{X}$, that minimizes $∥Y-H\hat{X}{∥}_{F}$, where ∥.∥_*F*_ is the Frobenius norm. In this case we suppose that the channel state information (CSI) is completely known at the receiver. A decoding error occurs if we choose E∈C, such that

$$∥R-\sqrt{E_{s}}{HE∥}_{F}<{∥R-\sqrt{E_{s}}HC∥}_{F}$$

for some *E* ≠ *C*. The pairwise error probability, denoted by *P*(*C* → *E*) in this case, is the probability of transmitting *C* and incorrectly decoding *E*.

Giving two matrices *C* and *C*′, we define the matrix **A**(*C*, *C*′) by **A**(*C*, *C*′) = (*C* − *C*′)(*C* − *C*′)*, where C* means the transpose conjugate of matrix *C*. Suppose that **A**(*C*, *E*) has rank *r* and non-null eigenvalues λ_1_, …, λ_*r*_. From [4], one has

$$P\left(C\to E|H\right)\leq \frac{1}{2}\prod_{j=1}^{n_{R}}\prod_{i=1}^{r}\text{exp}(-\lambda_{i}|\beta_{ji}{|}^{2}\frac{E_{s}}{4N_{0}})\;,$$ (1)

where, the coefficients *β*_*ij*_ are related with the terms *h*_*ij*_ of *H*. See [4, page 748].

One of the main results from [4] is a search criterion for STBC. It is currently known as **Rank and Determinant Criterion**. The expression in (1) is hard to manipulate. Using some approximations, it may be written in a simpler way and the error probability is given by

$$P\left(C\to E\right)\leq (\prod_{i=1}^{r}\lambda_{i}{)}^{-n_{R}}(\frac{E_{s}}{4N_{0}}{)}^{-rn_{R}}.$$ (2)

Therefore, good STBC for wireless channels, when *r* ⋅ *n*_*R*_ is small (≤ 4), must be searched to minimize (2). The criterion is given by:

- maximizing the minimum rank *r* of **A**(*C*, *C*′), on all pairs of distinct codewords;
- maximizing the product $\prod_{i=1}^{r}\lambda_{i}$ of eigenvalues of **A**(*C*, *C*′), between all pairs of distinct codewords.

Another important search criterion for STBC is also obtained from (1), when *r* ⋅ *n*_*R*_ > 4, established in [7]. Supposing the space-time code operates with reasonable SNR, after some approximations, the authors deduce that

$$P\left(C\to E\right)\leq \frac{1}{4}\text{exp}(-\frac{E_{s}}{4N_{0}}n_{R}\sum_{i=1}^{r}\lambda_{i}).$$ (3)

In this case, when *r* ⋅ *n*_*R*_ is large (≥ 4), the search of STBC must minimize (3). The limiting (3) shows that the error probability is dominated by codewords with minimum sum of eigenvalues of **A**(*C*, *E*), that is, *trace*(**A**(*C*, *E*)). Thus, the minimum sum of all eigenvalues of **A**(*C*, *E*) between all pairs of distinct codewords must be maximized. This criterion is called **Trace Criterion** and is given by:

- the minimum rank *r* of **A**(*C*, *C*′) over all pairs of distinct codewords so that *r n*_*R*_ ≥ 4;
- maximizing the minimum trace $\sum_{i=1}^{r}\lambda_{i}$ of **A**(*C*, *C*′) between all pairs of distinct codewords with minimum rank.

## 3 The matrix space where space-time block codes live in

In connection with the two criteria given, it must be observed that most works on STBC deal with the search of new codes. In [4], [7] and other works, a vector and a matrix are seen as the same object, that is, a matrix is a representation of a vector in a space $R^{n}$ or $C^{n}$. However, from a mathematical point of view, there exist deep analytical, algebraic and geometric differences when a codeword is seen as a vector $c=(c_{1}^{1}c_{1}^{2}\cdots c_{1}^{n_{T}}c_{2}^{1}c_{2}^{2}\cdots c_{2}^{n_{T}}\cdots c_{l}^{1}c_{l}^{2}\cdots c_{l}^{n_{T}})$ or a matrix *C* = (*c*_*ij*_)_*ij*_.

For the two criteria given, the two representations are used freely. The Frobenius norm is very useful, since the value ${∥M∥}_{F}^{2}$ of a matrix *M* is the square of Euclidian norm of *M*, seen as a vector. If we consider a convenient matrix space as a natural environment in which space-time codes, gaussian noise and fading matrices live in, and if this matrix space has enough rich analytic, algebraic and geometric structures, we will have powerful mathematical tools to manipulate the matrices. For instance, determinant, rank and trace, extensively used in space-time codes and MIMO research, are all operators on matrix spaces.

**Definition 3.1** *Let* M=M(m,n,C) *be the set of all m* × *n complex matrices*. *Under matrix addition and multiplication by complex numbers (scalars)*, M *is a vector space*. *Together with matrix multiplication*, *it is a matrix algebra*, *that is*, *an associative algebra of matrices*. *The spectral norm on* M *is the function* ∥.∥_2_: M → [0, ∞), *where*, *for a given* *A* ∈ M, *one has*

$${∥A∥}_{2}=\sqrt{\lambda_{max}\left(A^{*}A\right)}=\sigma_{max}\left(A\right)\;,$$

*where* λ_*max*_(*A*) *and σ*_*max*_(*A*) *are respectively*, *the largest eigenvalue and the largest singular value of A*. *The spectral norm has the following fundamental properties for all matrices A and B in* M *and all scalar α*:

i) ∥*A*∥_2_ ≥ 0
ii) ∥*A*∥_2_ = 0 ⇔ *A* = 0
iii) ∥*αA*∥_2_ = |*α*|∥*A*∥_2_
iv) ∥*A* + *B*∥_2_ ≤ ∥*A*∥_2_ + ∥*B*∥_2_.
v) ∥*AB*∥_2_ ≤ ∥*A*∥_2_∥*B*∥_2_

For a general approach on matrix norms and related results, see [47].

Space M, endowed with the spectral norm, is a Banach algebra. From property (iv), the following useful inequality is obtained,

$${|∥A∥}_{2}-{∥B∥}_{2}{|\leq ∥A-B∥}_{2}\;.$$

The equivalent definition can be proved

$${∥A∥}_{2}=\underset{x\neq 0}{\text{max}}\frac{{∥Ax∥}_{2}}{{∥x∥}_{2}}=\underset{{∥x∥}_{2}=1}{\text{max}}{∥Ax∥}_{2}\;.$$

We will also need the following relation between Frobenius and spectral norms.

**Proposition 3**.**1**

i) *For all matrix A* ∈ M *one has*
  $${∥A∥}_{2}\leq {∥A∥}_{F}\leq \sqrt{r}{∥A∥}_{2},$$ (4)
  *where r* ≤ min{*m*, *n*} *is the rank of A*.
ii) *If A is n* × *n*, *then*
  $$\left|trace\left(A\right)\right|\leq {n∥A∥}_{2}.$$

**Definition 3.2** *A space-time block code* (*STBC*) C *is a finite subset of* M.

Definition 3.2 is very generic. To obtain applicable STBC, subsets of M with good geometric and algebraic properties must be considered. Let C be a space-time block code. When a codeword C∈C is sent, the received signal is

$$R=\sqrt{E_{s}}HC+N.$$

As decoding rule, once *R* is received, our decoder will search the closest codeword E∈C of *R*, that is, *E*, such that ∥*R* − *E*∥_2_ is the minimum. Since ${∥A∥}_{2}^{2}=\lambda_{max}\left(AA^{*}\right)=\sigma_{max}^{2}\left(A\right)$ and *H* is known, if *E* is wrongly chosen, one has

$$\begin{array}{ccc} P(C\to E|H) & = & P(∥R-\sqrt{E_{s}}HE{∥}_{F}\leq ∥R-\sqrt{E_{s}}HC{∥}_{F})\\ & \leq & P(∥R-\sqrt{E_{s}}HE{∥}_{2}\leq \sqrt{n_{R}}∥R-\sqrt{E_{s}}HC{∥}_{2})\\ & = & P(∥\sqrt{E_{s}}HC+N-\sqrt{E_{s}}HE{∥}_{2}\leq \sqrt{n_{R}}∥\sqrt{E_{s}}HC+N-\sqrt{E_{s}}HC{∥}_{2})\\ & = & P(∥\sqrt{E_{s}}H\left(E-C\right)-N{∥}_{2}\;\leq \;\sqrt{n_{R}}∥N{∥}_{2})\\ & \leq & P(\sqrt{E_{s}}∥H\left(E-C\right){∥}_{2}-∥N{∥}_{2}\leq \;\sqrt{n_{R}}∥N{∥}_{2})\\ & = & P(\sqrt{E_{s}}∥H\left(E-C\right){∥}_{2}\leq \;\left(1+\sqrt{n_{R}}\right)∥N{∥}_{2})\\ & = & P(\frac{E_{s}}{{\left(1+\sqrt{n_{R}}\right)}^{2}}∥H\left(E-C\right){∥}_{2}^{2}\leq ∥N{∥}_{2}^{2})\\ & = & P(\frac{E_{s}}{{\left(1+\sqrt{n_{R}}\right)}^{2}}∥H\left(E-C\right){∥}_{2}^{2}\leq \lambda_{max}\left(NN^{*}\right))\;, \end{array}$$

where, in the first inequality, we used Proposition 3.1(*i*).

## 4 Random matrices and main results

In last section we deduced that

$$P\left(C\to E|H\right)\leq P(\frac{E_{s}}{{\left(1+\sqrt{n_{R}}\right)}^{2}}∥H\left(E-C\right){∥}_{2}^{2}\leq \lambda_{max}\left(NN^{*}\right))\;.$$

This implies that we need to find the pdf of the largest eigenvalue of NN*. To obtain this result, we have to use the theory of random matrices.

Random matrices were introduced by Eugene Wigner to model the nuclei of heavy atoms. In his works, Wigner realized that the eigenvalues distribution of a matrix with random Gaussian entries coincided with the statistics of fluctuations of the levels of heavy atoms, experimentally obtained. Thus, the pdf of eigenvalues of Random Matrices became an important object.

The set of all random variables *z* = *x* + *iy*, where *x* and *y* are iid *N*(*μ*, *σ*^2^), is denoted by $\tilde{N}\left(\mu ,\sigma^{2}\right)$. The following matrix sets are fundamental to the following.

**Definition 4.1** (*i*) *The complex Gaussian set* $\tilde{G}\left(m,n\right)$ *is the family of all m* × *n complex random matrices with independent and identically distributed* (*iid*) *elements which are* $\tilde{N}\left(0,\sigma^{2}\right)$.

(*ii*) *The complex Wishart set* $\tilde{W}\left(m,n\right)$ *is the family of all m* × *m complex random matrices*, which may be written in the form AA*, *where* $A\in \tilde{G}\left(m,n\right)$.

(*iii*) *The Gaussian Unitary Ensemble GUE is the set of all symmetric m* × *m complex matrices with* (*iid*) *elements that are N*(0, 1/4) *in the upper-triangle and iid elements that are N*(0, 1/2) *on the diagonal*.

Now, considering $\tilde{G}\left(m,n\right)$, where the elements in the matrices are $\tilde{N}\left(0,\sigma^{2}\right)$, one has the following result from [48].

**Theorem 4.1** *Given* $\tilde{M}=\tilde{A}\tilde{A^{*}}\in \tilde{W}\left(m,n\right)$, *where* $\tilde{A}\in \tilde{G}\left(m,n\right)$, *suppose* λ_1_ ≥ λ_2_ ≥ ⋯ ≥ λ_*m*−1_ ≥ λ_*m*_ ≥ 0 *are the eigenvalues of* $\tilde{M}$. *Then*, *the joint pdf of the eigenvalues of* $\tilde{M}$ *is*

$$\tilde{f}\left(\lambda_{1},\lambda_{2},\cdots ,\lambda_{m}\right)={\tilde{K}}_{n,m}\text{exp}(-\frac{1}{2\sigma^{2}}\sum_{i=1}^{m}\lambda_{i})\prod_{i=1}^{m}\lambda_{i}^{n-m}\prod_{i<j}{\left(\lambda_{i}-\lambda_{j}\right)}^{2},$$

*where*

$${\tilde{K}}_{n,m}^{-1}={\left(2\sigma^{2}\right)}^{mn}\prod_{i=1}^{m}\Gamma (n-i+1)\Gamma (m-i+1).$$ (5)

Now, we want the pdf of the largest eigenvalue of a complex Wishart matrix. Results found in literature, for instance, in [49] and [50], are not easy to manipulate. Thus, an approximation to the pdf will be obtained. We begin with the following bound.

**Theorem 4.2** *If* $\tilde{M}\in \tilde{W}\left(m,n\right)$, *then* $f_{\lambda_{max}}\left(\lambda \right)$ *satisfies*

$$\begin{array}{ccc} f_{\lambda_{max}}\left(\lambda \right) & \leq & \frac{{\tilde{K}}_{n,m}}{{\tilde{K}}_{n-1,m-1}}\lambda^{n+m-2}\text{exp}(-\frac{\lambda }{2\sigma^{2}})\\ & = & \frac{{\left(2\sigma^{2}\right)}^{-n-m+1}}{\Gamma (n)\Gamma (m)}\lambda^{n+m-2}\text{exp}(-\frac{\lambda }{2\sigma^{2}}). \end{array}$$

**Proof**. From Theorem 4.1, one has

$$\begin{array}{c} \begin{array}{c} \tilde{f}\left(\lambda_{1},\lambda_{2},\cdots ,\lambda_{m}\right)={\tilde{K}}_{n,m}\text{exp}(-\frac{\lambda_{1}}{2\sigma^{2}})\lambda_{1}^{n-m}\text{exp}(\sum_{i=2}^{m}-\frac{\lambda_{i}}{2\sigma^{2}})\\ \times \prod_{i=2}^{m}({\left(\lambda_{1}-\lambda_{i}\right)}^{2}\cdot \lambda_{i}^{n-m})\prod_{i<j}{\left(\lambda_{i}-\lambda_{j}\right)}^{2}. \end{array} \end{array}$$

Thus,

$$\begin{array}{ccc} f_{\lambda_{max}}\left(\lambda \right) & = & {\tilde{K}}_{n,m}\text{exp}(-\frac{\lambda }{2\sigma^{2}})\lambda^{n-m}\int_{R_{2}}\text{exp}(\sum_{i=2}^{m}-\frac{\lambda_{i}}{2\sigma^{2}})\\ & & \times \prod_{i=2}^{m}({\left(\lambda -\lambda_{i}\right)}^{2}\cdot \lambda_{i}^{n-m})\prod_{i<j}{\left(\lambda_{i}-\lambda_{j}\right)}^{2}d\lambda_{i}, \end{array}$$

where *R*_2_ = {(λ_2_, λ_3_, ⋯, λ_*m*_): λ_2_ ∈ [0, λ]; λ_*i*_ ∈ [0, λ_*i*−1_], *i* ∈ {3, ⋯, *m*}}. Since 0 ≤ λ − λ_*i*_ ≤ λ, then 0 ≤ (λ − λ_*i*_)^2^ ≤ λ^2^, and this may be bounded above by

$$\begin{array}{ccc} f_{\lambda_{max}}\left(\lambda \right) & \leq & {\tilde{K}}_{n,m}\text{exp}(-\frac{\lambda }{2\sigma^{2}})\lambda^{n-m}\int_{R}\text{exp}(\sum_{i=2}^{m}-\frac{\lambda_{i}}{2\sigma^{2}})\\ & & \times \prod_{i=2}^{m}(\lambda^{2}\cdot \lambda_{i}^{n-m})\prod_{i<j}{\left(\lambda_{i}-\lambda_{j}\right)}^{2}d\lambda_{i}\\ & \leq & {\tilde{K}}_{n,m}\text{exp}(-\frac{\lambda }{2\sigma^{2}})\lambda^{n+m-2}\int_{R_{2}}\text{exp}(\sum_{i=2}^{m}-\frac{\lambda_{i}}{2\sigma^{2}})\\ & & \times \prod_{i=2}^{m}(\lambda_{i}^{n-m})\prod_{i<j}{\left(\lambda_{i}-\lambda_{j}\right)}^{2}d\lambda_{i}, \end{array}$$

where *R*_2_ = {(λ_2_, ⋯, λ_*m*_): λ_2_ ∈ [0, ∞]; λ_*i*_ ∈ [0, λ_*i*−1_], *i* ∈ {3, ⋯, *m*}}. From Theorem 4.1, we have

$${\tilde{K}}_{n-1,m-1}^{-1}=\int_{R_{2}}\text{exp}(\sum_{i=2}^{m}\frac{-\lambda_{i}}{2\sigma^{2}})\prod_{i=2}^{m}\lambda_{i}^{n-m}\prod_{i<j}{\left(\lambda_{i}-\lambda_{j}\right)}^{2}d\lambda_{i}\;,$$ (6)

and, substituting (6) in the limiting of $f_{\lambda_{max}}\left(\lambda \right)$, one has

$$f_{\lambda_{max}}\left(\lambda \right)\leq \frac{{\tilde{K}}_{n,m}}{{\tilde{K}}_{n-1,m-1}}\lambda^{n+m-2}\text{exp}(-\frac{\lambda }{2\sigma^{2}}).$$ (7)

Finally, using the expression of ${\tilde{K}}_{n,m}$ in Eq (7), one has

$$\begin{array}{ccc} f_{\lambda_{max}}\left(\lambda \right) & \leq & \frac{{\left(2\sigma^{2}\right)}^{-n-m+1}}{\Gamma (n)\Gamma (m)}\lambda^{n+m-2}\text{exp}(-\frac{\lambda }{2\sigma^{2}})\;, \end{array}$$

which concludes the proof.

Now, with the bound above, we may build our approximation function. Since

$$\int_{0}^{\infty }\frac{{\left(2\sigma^{2}\right)}^{-n-m+1}}{\Gamma (n)\Gamma (m)}\lambda^{n+m-2}\text{exp}(-\frac{\lambda }{2\sigma^{2}})d\lambda =\frac{\Gamma (n+m-1)}{\Gamma (n)\Gamma (m)}\;,$$

normalizing the bound of Theorem 4.2, we define the function

$$g\left(\lambda \right)=\frac{{\left(2\sigma^{2}\right)}^{-n-m+1}}{\Gamma (n+m-1)}\lambda^{n+m-2}\text{exp}(-\frac{\lambda }{2\sigma^{2}})\;.$$ (8)

**Fig 1:**
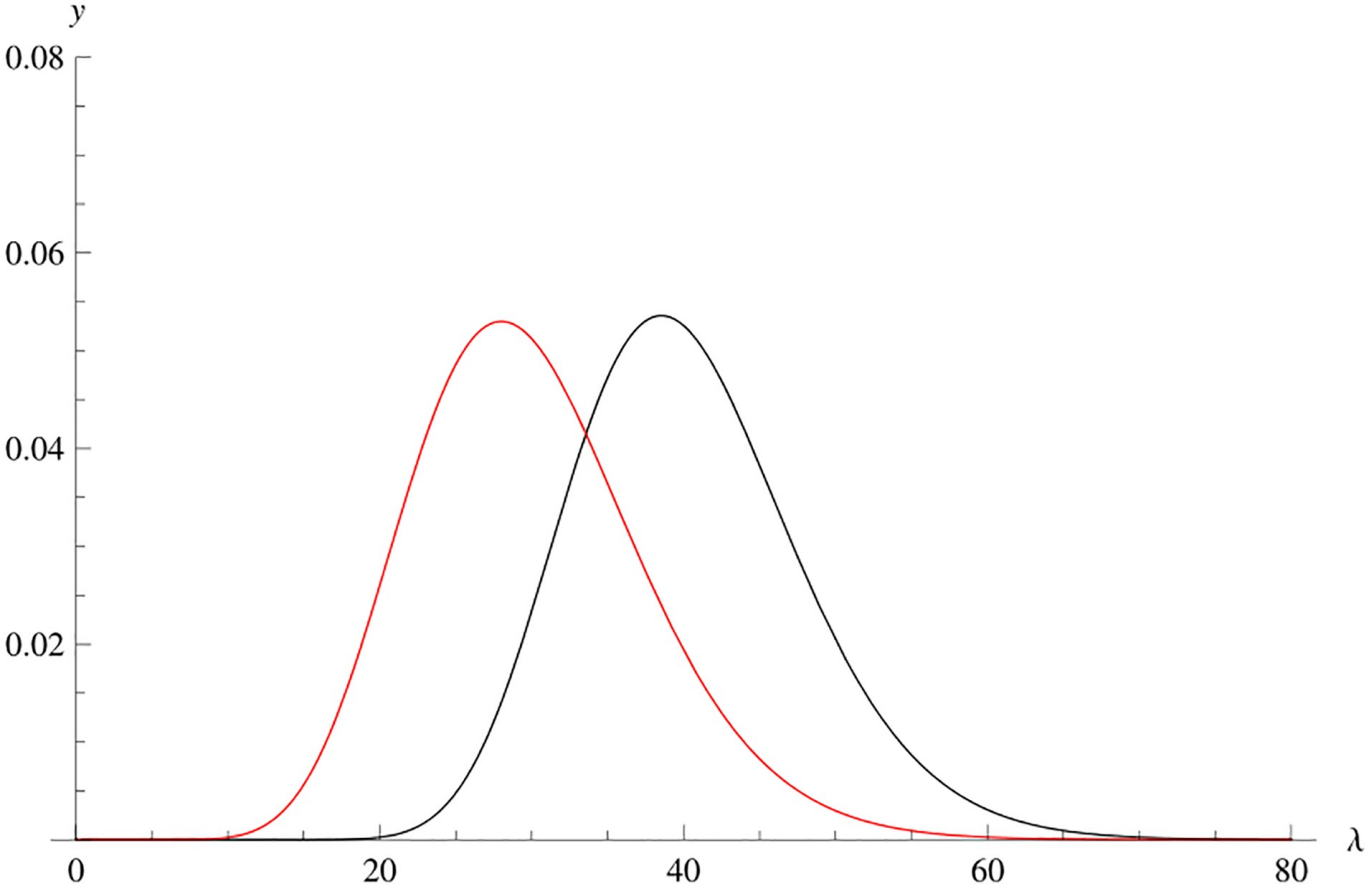
Graphs of exact pdf $f_{\lambda_{max}}\left(\lambda \right)$ and of *g*(λ) for $\tilde{W}\left(3,13\right)$.

Then, *g* is a pdf on [0, ∞). Using an algebraic computer program, $f_{\lambda_{max}}\left(\lambda \right)$ may be plotted for all cases of *m* and *n*. Comparing cases of *g* with $f_{\lambda_{max}}$, for the same pair (*m*, *n*), it may be seen that a translation of *g*(λ) is a good approximation to $f_{\lambda_{max}}\left(\lambda \right)$. Fig 1 provides an example.

**Fig 2:**
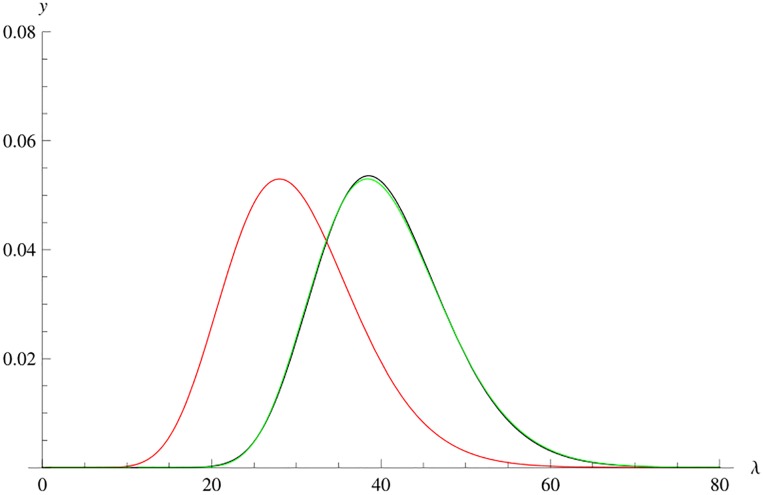
Graphs of $f_{\lambda_{max}}\left(\lambda \right)$, *g*(λ) and the translation with *d*_1_ = 10.4 for $\tilde{W}\left(3,13\right)$.

Thus, a constant *d*_1_ = *d*_1_(*m*, *n*) must be found, such that the translation of *g*(λ), given by

$$\phi \left(\lambda \right)=\{\begin{array}{cc} 0\;, & 0\leq \lambda <d_{1}\\ \frac{{\left(2\sigma^{2}\right)}^{-n-m+1}\cdot (\lambda -d_{1}{)}^{n+m-2}}{\Gamma (n+m-1)}\text{exp}(-\frac{\left(\lambda -d_{1}\right)}{2\sigma^{2}})\;, & \lambda \geq d_{1} \end{array},$$

is an approximation to $f_{\lambda_{max}}\left(\lambda \right)$. Fig 2 shows the graphs of $f_{\lambda_{max}}\left(\lambda \right)$, *g*(λ) and *ϕ*(λ − 10.4) for $\tilde{W}\left(3,13\right)$.

Table 1 shows the exact pdf $f_{\lambda_{max}}\left(\lambda \right)$ for some cases and the translations of *g*(λ) which fit better, such that *ϕ*(λ) is the best approximation. Data were obtained by trial and error to minimize the distance

$$\int_{0}^{\infty }|f_{\lambda_{max}}\left(t\right)-\phi \left(t\right)|dt,$$

between $f_{\lambda_{max}}\left(\lambda \right)$ and *ϕ*(λ). Table 1 also presents the maximum point of *ϕ*(*t*). For simplicity, in Tables 1 and 2, it is assumed that *σ*^2^ = 1. Since *ϕ*(λ) is a translation of *g*(λ), they have the same maximum.

**Table 1:** Better translation and its maximum point.

$\tilde{W}\left(m,n\right)$	better translation	maximum point of the better translation
$\tilde{W}\left(2,2\right)$	1.0	5.0
$\tilde{W}\left(2,3\right)$	1.7	7.7
$\tilde{W}\left(2,4\right)$	2.4	10.4
$\tilde{W}\left(2,15\right)$	6.7	36.7
$\tilde{W}\left(2,22\right)$	8.7	52.7
$\tilde{W}\left(3,3\right)$	3.2	11.2
$\tilde{W}\left(3,5\right)$	5.1	17.1
$\tilde{W}\left(3,13\right)$	10.4	38.4
$\tilde{W}\left(3,25\right)$	16.0	68
$\tilde{W}\left(4,4\right)$	5.5	17.5
$\tilde{W}\left(4,7\right)$	9.0	27
$\tilde{W}\left(4,13\right)$	14.0	44
$\tilde{W}\left(5,5\right)$	8.4	24.4
$\tilde{W}\left(5,9\right)$	13.2	37.2

**Table 2:** Maximum points and their differences.

$\tilde{W}\left(m,n\right)$	maximum point $f_{\lambda_{max}}\left(\lambda \right)$	maximum point of *g*(λ)	difference
$\tilde{W}\left(2,2\right)$	5.11	4.0	1.1
$\tilde{W}\left(2,3\right)$	7.89	6.0	1.89
$\tilde{W}\left(2,4\right)$	10.53	8.0	2.53
$\tilde{W}\left(2,15\right)$	36.7	30.0	6.7
$\tilde{W}\left(2,22\right)$	52.58	44.0	12.58
$\tilde{W}\left(3,3\right)$	11.22	8.0	3.22
$\tilde{W}\left(3,5\right)$	17.24	12.0	5.24
$\tilde{W}\left(3,13\right)$	38.52	28.0	10.52
$\tilde{W}\left(3,25\right)$	67.79	52.0	15.79
$\tilde{W}\left(4,4\right)$	17.76	12.0	5.76
$\tilde{W}\left(4,7\right)$	27.15	18.0	9.15
$\tilde{W}\left(4,13\right)$	44.06	30.0	14.06
$\tilde{W}\left(5,5\right)$	24.53	16.0	8.53
$\tilde{W}\left(5,9\right)$	37.6	24.0	13.6

It is not possible to find analytically the maximum point of $f_{\lambda_{max}}\left(\lambda \right)$. However, the maximum point of *g*(λ) may be found. Given the maximum point of *g*(λ), we must determine the constant *d*_1_ = *d*_1_(*m*, *n*). Supposing *σ*^2^ = 1, the maximum point of *g*(λ) is λ_0_ = 2(*n* + *m* − 2). Thus, λ_0_ + *d*_1_(*m*, *n*) = 2(*n* + *m* − 2) + *d*_1_(*m*, *n*) must coincide with the maximum point of $f_{\lambda_{max}}\left(\lambda \right)$. Let *h*(*m*, *n*) be the maximum point of $f_{\lambda_{max}}\left(\lambda \right)$, then

$$h\left(m,n\right)=2\left(n+m-2\right)+d_{1}\left(m,n\right)$$

and

$$d_{1}\left(m,n\right)=h\left(m,n\right)-2\left(n+m-2\right).$$

Using data from Table 2, an expression to *d*_1_(*m*, *n*) will be obtained by the least squares method. Plotting the data of Table 2, the function describing *d*_1_(*m*, *n*) may be seen as a plane and its equation is given by

$$d_{1}\left(m,n\right)=am+bn+c\;,$$

where

$$d_{1}\left(m_{i},n_{i}\right)=\mu_{i},$$

and *μ*_*i*_ are the data of the third column of Table 2. Thus, we must find the constants *a*, *b* and *c* by minimizing:

$$F\left(a,b,c\right)=\sum_{i=1}^{14}{\left(am_{i}+bn_{i}+c-\mu_{i}\right)}^{2}.$$

Then, we need to solve the equation ∇*F*(*a*, *b*, *c*) = 0, given by

$$\begin{array}{c} \left\{ \begin{array}{c} 2\sum_{i=1}^{14}\left(am_{i}+bn_{i}+c-\mu_{i}\right)\left(m_{i}\right)=0\\ 2\sum_{i=1}^{14}\left(am_{i}+bn_{i}+c-\mu_{i}\right)\left(n_{i}\right)=0\\ 2\sum_{i=1}^{14}\left(am_{i}+bn_{i}+c-\mu_{i}\right)\left(1\right)=0 \end{array}.\right. \end{array}$$

From Table 2, one has

$$\begin{array}{c} \left\{ \begin{array}{c} 154a+396b+44c=380.44\\ 396a+1906b+130c=1397.54\\ 44a+130b+14c=110.67 \end{array},\right. \end{array}$$

and the solution is given by

{a,b,c}={2.53573,0.574893,-5.40273}.

Therefore, the translation of *g*(λ) is

$$d_{1}\left(m,n\right)=2.53573m+0.574893n-5.40273.$$ (9)

Putting together the results, one has

**Theorem 4.3** *An approximation to the pdf of the largest eigenvalue of a Wishart matrix* $N_{n_{R}\times l}N_{l\times n_{R}}^{*}$, *with variance σ*^2^ = *N*_0_/2, *is the given by the pdf*

$$\begin{array}{c} \phi \left(t\right)=\{\begin{array}{cc} 0 & ,0\leq t<d_{1}\\ (\frac{t-d_{1}}{N_{0}}{)}^{l+n_{R}-2}\frac{\text{exp}(-\frac{\left(t-d_{1}\right)}{N_{0}})}{\Gamma \left(l+n_{R}-1\right)\cdot N_{0}} & ,t\geq d_{1} \end{array}, \end{array}$$

*where d*_1_ = *d*_1_(*n*_*R*_, *l*) = 2.53573*n*_*R*_ + 0.574893*l* − 5.40273.

In what follows in the text, we are considering *d*_1_ = *d*_1_(*n*_*R*_, *l*), where *n*_*R*_ × *l* is the dimension of matrix *N*.

**Remark 1**. Table 3 shows values of *d*_1_(*m*, *n*), which may be compared with those in Tables 1 and 2. We have

$$\bar{\mu }=\frac{1}{14}\sum_{i=1}^{14}\mu_{i}=7.905\;.$$

Then, the *total variation* is

$$\sum_{i=1}^{14}{\left(\mu_{i}-\bar{\mu }\right)}^{2}=309.68,$$

and the *explained variation* is

$$\sum_{i=1}^{14}{\left(d_{1}\left(m_{i},n_{i}\right)-\bar{\mu }\right)}^{2}=290.364.$$

Therefore, the *coefficient of determination* is **R**^2^ = 290.364/309.68 = 0.953994, implying that the model explains the observed values with 95% of confidence.

**Table 3:** Values for the translation.

*d*_1_(*m*, *n*) = 2.53573*m* + 0.574893*n* − 5.40273	value
*d*_1_(2, 2)	0.8185
*d*_1_(2, 3)	1.3934
*d*_1_(2, 4)	1.9683
*d*_1_(2, 15)	8.2921
*d*_1_(2, 22)	12.3164
*d*_1_(3, 3)	3.9291
*d*_1_(3, 5)	5.0789
*d*_1_(3, 13)	9.6780
*d*_1_(3, 25)	16.5768
*d*_1_(4, 4)	7.0397
*d*_1_(4, 7)	8.7644
*d*_1_(4, 13)	12.2138
*d*_1_(5, 5)	10.1504
*d*_1_(5, 9)	12.45

## 5 A new criterion to search STBC

Up to the present, the use of random matrices to obtain a search criterion of STBC for MIMO channels is unknown in the literature. Results in this section will establish one. From Section 3, we need to calculate

$$P(\frac{E_{s}}{{\left(1+\sqrt{n_{R}}\right)}^{2}}∥H\left(E-C\right){∥}_{2}^{2}\;\leq \;\lambda_{max}\left(NN^{*}\right))\;.$$ (10)

Since

$$P\left(a\leq \lambda_{max}\left(NN^{*}\right)\right)=\int_{a}^{\infty }f_{\lambda_{max}}\left(\lambda \right)d\lambda ,$$

where $f_{\lambda_{max}}\left(\lambda \right)$ is the pdf of the largest eigenvalue of a Wishart matrix. Theorem 4.3 shows

$$P\left(a\leq \lambda_{max}\left(NN^{*}\right)\right)=\int_{a}^{\infty }\phi \left(t\right)dt.$$

If 0 ≤ *a* ≤ *d*_1_, then

$$\int_{a}^{\infty }\phi \left(t\right)dt=1.$$

Eq (10) is the probability of the maximum likelihood decoder, when receiving *R* choose wrongly *E*, if *C* was sent. When 0 ≤ *a* < *d*_1_, an error occurred. On the other hand, if *a* ≥ *d*_1_,

$$\begin{array}{ccc} \int_{a}^{\infty }\phi \left(t\right)dt & = & \int_{a}^{\infty }\frac{{\left(N_{0}\right)}^{-l-n_{R}+1}\cdot (t-d_{1}{)}^{\left(l+n_{R}-2\right)}}{\Gamma (l+n_{R}-1)}\\ & & \times \text{exp}(-\frac{t-d_{1}}{N_{0}})dt\\ & = & \frac{1}{\Gamma (l+n_{R}-1)}\Gamma (l+n_{R}-1,\frac{a-d_{1}}{N_{0}}). \end{array}$$

Hence,

$$\begin{array}{c} P(a\leq \lambda_{max}\left(NN^{*}\right))=\{\begin{array}{cc} 1 & ,0\leq a\leq d_{1}\\ \frac{\Gamma (l+n_{r}-1,\frac{a-d_{1}}{N_{0}})}{\Gamma (l+n_{r}-1)}\; & ,d_{1}<a \end{array}, \end{array}$$

where *d*_1_ = *d*_1_(*n*_*R*_, *l*) is given by (9). Therefore, we proved the following:

**Theorem 5.1** *In a MIMO communication channel*, *where the codeword C was sent*, *if the maximum likelihood decoder is endowed with the spectral norm*, *the error probability of received signal be decoded by the codeword E*, *given that H is known*, *is*

$$\begin{array}{c} P\left(C\to E|H\right)=P(\frac{E_{s}}{{\left(1+\sqrt{n_{R}}\right)}^{2}}∥H\left(E-C\right){∥}_{2}^{2}\leq \lambda_{max}\left(NN^{*}\right))\\ \;=\{\begin{array}{cc} 1 & ,0\leq \frac{E_{s}∥H\left(E-C\right){∥}_{2}^{2}}{{\left(1+\sqrt{n_{R}}\right)}^{2}}\leq d_{1}\\ \frac{\Gamma (l+n_{r}-1,\frac{E_{s}∥H\left(E-C\right){∥}_{2}^{2}-{\left(1+\sqrt{n_{R}}\right)}^{2}d_{1}}{{\left(1+\sqrt{n_{R}}\right)}^{2}N_{0}})}{\Gamma (l+n_{r}-1)} & ,d_{1}<\frac{E_{s}∥H\left(E-C\right){∥}_{2}^{2}}{{\left(1+\sqrt{n_{R}}\right)}^{2}} \end{array}, \end{array}$$

*where d*_1_ = *d*_1_(*n*_*R*_, *l*) = 2.53573*n*_*r*_ + 0.574893*l* − 5.40273.

Up to now we are supposing that *H* is known, that is, the statistics of *H* are known. Now, we want to calculate the mean in *H*, that is,

$$P\left(C\to E\right)=\int_{Dom\;p(H)}P\left(C\to E|H\right)p\left(H\right)dH,$$ (11)

where *p*(*H*) is a pdf of the matrix *H*.

Theorem 5.1 shows that the term ∥*H*(*E* − *C*)∥_2_ is our main concern, since we need more information on the term $\Gamma (l+n_{r}-1,\frac{E_{s}∥H\left(E-C\right){∥}_{2}^{2}-{\left(1+\sqrt{n_{R}}\right)}^{2}d_{1}}{{\left(1+\sqrt{n_{R}}\right)}^{2}N_{0}})/\Gamma (l+n_{r}-1)$.

**Fig 3:**
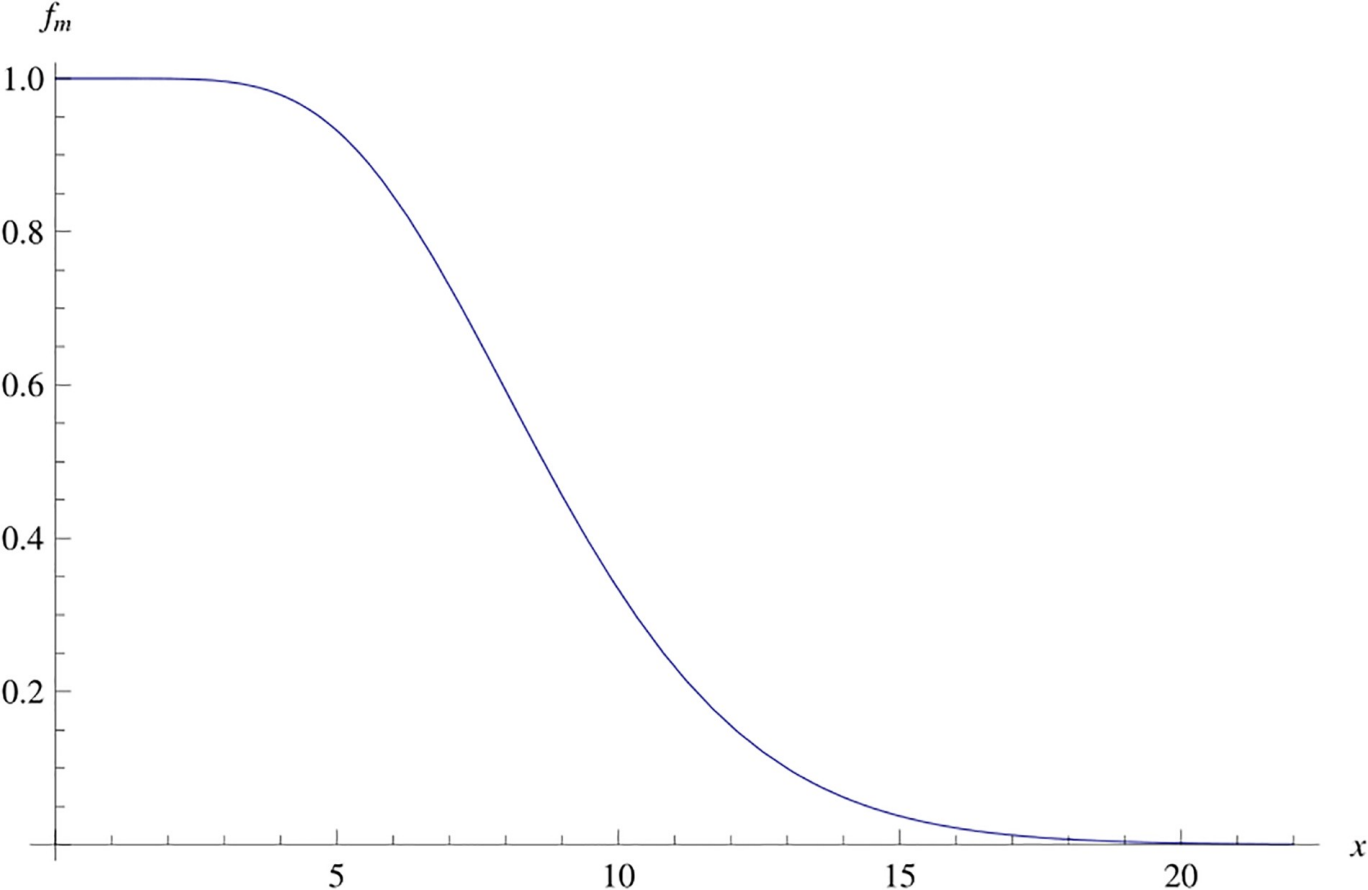
Graph of *f*_*m*_(*x*) for *m* = 9.

Define *f*_*m*_(*x*) = Γ(*m*, *x*)/Γ(*m*) for *m* > 0 fixed and *x* ≥ 0. A typical example is shown in Fig 3. We know that *f*_*m*_(*x*) is a fast decreasing function, such that 0 < *f*_*m*_(*x*) ≤ 1, lim_*x*→∞_ *f*_*m*_(*x*) = 0 and lim_*m*→∞_ *f*_*m*_(*x*) = 1, for all *x*.

Let $t=∥H{∥}_{2}^{2}$ and $c=∥\left(E-C\right){∥}_{2}^{2}$, then

$$\begin{array}{c} \frac{E_{s}∥H{∥}_{2}^{2}∥\left(E-C\right){∥}_{2}^{2}}{{\left(1+\sqrt{n_{R}}\right)}^{2}N_{0}}-\frac{d_{1}}{N_{0}}=\frac{E_{s}tc}{{\left(1+\sqrt{n_{R}}\right)}^{2}N_{0}}-\frac{d_{1}}{N_{0}}=\frac{E_{s}tc-{\left(1+\sqrt{n_{R}}\right)}^{2}d_{1}}{{\left(1+\sqrt{n_{R}}\right)}^{2}N_{0}}\;, \end{array}$$

and from property (*v*) of spectral norm, one has

$$\begin{array}{ccc} d_{1} & < & \frac{E_{s}∥H\left(E-C\right){∥}_{2}^{2}}{{\left(1+\sqrt{n_{R}}\right)}^{2}}\\ & \leq & \frac{E_{s}∥H{∥}_{2}^{2}\cdot ∥\left(E-C\right){∥}_{2}^{2}}{{\left(1+\sqrt{n_{R}}\right)}^{2}}\;. \end{array}$$

Thus, $\frac{{\left(1+\sqrt{n_{R}}\right)}^{2}d_{1}}{E_{s}c}<t<\infty$ and from the behavior of *f*_*m*_(*x*), it will be enough to assume the following

$$\begin{array}{ccc} P(C\to E|H) & \approx & \left\{ \begin{array}{cc} 1 & ,0\leq t\leq \frac{{\left(1+\sqrt{n_{R}}\right)}^{2}d_{1}}{E_{s}c}\\ \frac{\Gamma (l+n_{r}-1,\frac{E_{s}tc-{\left(1+\sqrt{n_{R}}\right)}^{2}\cdot d_{1}}{{\left(1+\sqrt{n_{R}}\right)}^{2}N0})}{\Gamma (l+n_{r}-1)} & ,\frac{{\left(1+\sqrt{n_{R}}\right)}^{2}d_{1}}{E_{s}c}<t<\infty \end{array}\;\;\right. \end{array}$$ (12)

The elements of $H_{n_{R}\times n_{T}}$ are Gaussian random complex variables with mean zero and variance 1/2. From Theorem 4.3, the pdf of the largest eigenvalue of $H_{n_{R}\times n_{T}}H_{n_{T}\times n_{R}}^{*}$ is given by

$$\begin{array}{c} \psi \left(t\right)=\{\begin{array}{cc} 0 & ,t<d_{2}\\ \frac{{\left(t-d_{2}\right)}^{n_{T}+n_{R}-2}}{\Gamma (n_{T}+n_{R}-1)}\text{exp}(-t+d_{2}) & ,d_{2}\leq t \end{array}, \end{array}$$ (13)

where *d*_2_ = *d*_2_(*n*_*R*_, *n*_*T*_) = 2.53573*n*_*R*_ + 0.574893*n*_*T*_ − 5.402373. Prior to proving one of our main results, we need the following proposition.

**Proposition 5.1** *We have that*

$$\begin{array}{c} \int_{d_{2}}^{\infty }\text{exp}(-\frac{E_{s}ct}{{\left(1+\sqrt{n_{R}}\right)}^{2}N_{0}}){\left(E_{s}ct-{\left(1+\sqrt{n_{R}}\right)}^{2}d_{1}\right)}^{i}{\left(t-d_{2}\right)}^{n_{T}+n_{R}-2}\text{exp}\left(-t\right)dt\\ \;=\sum_{j=0}^{i}{(\left(-1\right)}^{j}(\begin{array}{c} i\\ j \end{array}){\left(E_{s}c\right)}^{i-j}{\left(\frac{{\left(1+\sqrt{n_{R}}\right)}^{2}N_{0}+E_{s}c}{{\left(1+\sqrt{n_{R}}\right)}^{2}N_{0}}\right)}^{j-i-n_{T}-n_{R}+1}\\ \;\times {\left({\left(1+\sqrt{n_{R}}\right)}^{2}d_{1}\right)}^{j}\Gamma \left(-j+i+n_{T}+n_{R}-1\right)\text{exp}(-\frac{d_{2}}{{\left(1+\sqrt{n_{R}}\right)}^{2}N_{0}}\left(E_{s}c+{\left(1+\sqrt{n_{R}}\right)}^{2}N_{0}\right))\\ \;\times 1F1(j-i,j-i-n_{T}-n_{R}+2,\frac{d_{2}}{{\left(1+\sqrt{n_{R}}\right)}^{2}N_{0}}\left(E_{s}c+{\left(1+\sqrt{n_{R}}\right)}^{2}N_{0}\right))), \end{array}$$

*where* 1*F*1(*a*, *b*; *z*) *is the confluent hypergeometric function*.

**Proof**. From Newton’s binomial formula, one has ${\left(E_{s}ct-{\left(1+\sqrt{n_{R}}\right)}^{2}d_{1}\right)}^{i}=\sum_{j=0}^{i}{\left(-1\right)}^{j}(\begin{array}{c} i\\ j \end{array}){\left(E_{s}ct\right)}^{i-j}{\left({\left(1+\sqrt{n_{R}}\right)}^{2}d_{1}\right)}^{j}$. Then, we need to calculate

$$\begin{array}{c} \sum_{j=0}^{i}{\left(-1\right)}^{j}(\begin{array}{c} i\\ j \end{array}){\left(E_{s}c\right)}^{i-j}\int_{d_{2}}^{\infty }\text{exp}(-\frac{t(E_{s}c+{\left(1+\sqrt{n_{R}}\right)}^{2}N_{0})}{{\left(1+\sqrt{n_{R}}\right)}^{2}N_{0}})\\ \times {\left({\left(1+\sqrt{n_{R}}\right)}^{2}d_{1}\right)}^{j}{\left(t\right)}^{i-j}{\left(t-d_{2}\right)}^{n_{T}+n_{R}-2}dt. \end{array}$$

Since

$$\begin{array}{ccc} & & \int_{d_{2}}^{\infty }\text{exp}(-\frac{t(E_{s}c+{\left(1+\sqrt{n_{R}}\right)}^{2}N_{0})}{{\left(1+\sqrt{n_{R}}\right)}^{2}N_{0}}){\left(t\right)}^{i-j}{\left(t-d_{2}\right)}^{n_{T}+n_{R}-2}dt\\ & = & {\left(\frac{{\left(1+\sqrt{n_{R}}\right)}^{2}N_{0}+E_{s}c}{{\left(1+\sqrt{n_{R}}\right)}^{2}N_{0}}\right)}^{1-i+j-n_{R}-n_{T}}\Gamma \left(-1+i-j+n_{R}+n_{T}\right)\\ & & \times \text{exp}(-\frac{d_{2}\left(E_{s}c+{\left(1+\sqrt{n_{R}}\right)}^{2}N_{0}\right)}{{\left(1+\sqrt{n_{R}}\right)}^{2}N_{0}})\\ & & \times 1F1[-i+j,2-i+j-n_{R}-n_{T},\frac{d_{2}\left(E_{s}c+{\left(1+\sqrt{n_{R}}\right)}^{2}N_{0}\right)}{{\left(1+\sqrt{n_{R}}\right)}^{2}N_{0}}], \end{array}$$

the result follows.

Now, we have one of the most important result.

**Theorem 5.2** *In a MIMO communication channel*, *given that the codeword C was sent*, *if the decoder is endowed with the spectral norm*, *the error probability of received signal be decoded by the codeword E*, *is*

$$\begin{array}{ccc} P\left(C\to E\right)=\sum_{i=0}^{l+nr-2}\sum_{j=0}^{i}\sum_{k=0}^{i-j}\{{\left(\frac{1}{{\left(1+\sqrt{n_{R}}\right)}^{2}N_{0}}\right)}^{j+k-n_{T}-n_{R}+1}\frac{{\left(-1\right)}^{j}}{i!\cdot k!} & & \\ \;\times (\begin{array}{c} i\\ j \end{array}){\left(n_{T}+n_{R}-1\right)}_{i-j}\frac{{\left(j-i\right)}_{k}}{{\left(j-i-n_{T}-n_{R}+2\right)}_{k}}{\left(E_{s}c\right)}^{i-j}\\ \;\times {\left({\left(1+\sqrt{n_{R}}\right)}^{2}N_{0}+E_{s}c\right)}^{j-i+k-n_{T}-n_{R}+1}\}\\ \;\times {\left({\left(1+\sqrt{n_{R}}\right)}^{2}d_{1}\right)}^{j}d_{2}^{k}\text{exp}(\frac{-E_{s}cd_{2}+{\left(1+\sqrt{n_{R}}\right)}^{2}d_{1}}{{\left(1+\sqrt{n_{R}}\right)}^{2}N_{0}}), \end{array}$$ (14)

*where* (*x*)_*k*_ *represents the Pochhammer symbol*, *defined by* (*x*)_*k*_ = *x*(*x* − 1)(*x* − 2) ⋯ (*x* − *k* + 1).

**Proof**. From (12) and (13), the probability $P\left(C\to E\right)=\int_{Dom\;\psi (t)}P\left(C\to E|H\right)\psi \left(t\right)dt$, is given by

$$\int_{d_{2}}^{\infty }\frac{\Gamma (l+n_{R}-1,\frac{E_{s}ct-{\left(1+\sqrt{n_{R}}\right)}^{2}d_{1}}{{\left(1+\sqrt{n_{R}}\right)}^{2}N_{0}})}{\Gamma (l+n_{R}-1)}[\frac{{\left(t-d_{2}\right)}^{n_{T}+n_{R}-2}\text{exp}(-t+d_{2})}{\Gamma (n_{T}+n_{R}-1)}]dt.$$ (15)

Since

$$\frac{\Gamma (l+n_{R}-1,\frac{E_{s}ct-{\left(1+\sqrt{n_{R}}\right)}^{2}d_{1}}{{\left(1+\sqrt{n_{R}}\right)}^{2}N_{0}})}{\Gamma (l+n_{R}-1)}=\sum_{i=0}^{l+nr-2}\frac{{\left(\frac{E_{s}ct-{\left(1+\sqrt{n_{R}}\right)}^{2}d_{1}}{{\left(1+\sqrt{n_{R}}\right)}^{2}N_{0}}\right)}^{i}}{i!}\text{exp}(-\frac{E_{s}ct-{\left(1+\sqrt{n_{R}}\right)}^{2}d_{1}}{{\left(1+\sqrt{n_{R}}\right)}^{2}N_{0}}),$$ (16)

one has

$$\begin{array}{c} \int_{d_{2}}^{\infty }(\frac{\Gamma (l+n_{R}-1,\frac{E_{s}ct-{\left(1+\sqrt{n_{R}}\right)}^{2}d_{1}}{{\left(1+\sqrt{n_{R}}\right)}^{2}N_{0}})}{\Gamma (l+n_{R}-1)})[\frac{{\left(t-d_{2}\right)}^{n_{T}+n_{R}-2}}{\Gamma (n_{T}+n_{R}-1)}\text{exp}(-t+d_{2})]dt\\ \;=\int_{d_{2}}^{\infty }(\text{exp}(\frac{-E_{s}ct+{\left(1+\sqrt{n_{R}}\right)}^{2}d_{1}}{{\left(1+\sqrt{n_{R}}\right)}^{2}N_{0}})\sum_{i=0}^{l+nr-2}\frac{{\left(\frac{E_{s}ct-{\left(1+\sqrt{n_{R}}\right)}^{2}d_{1}}{{\left(1+\sqrt{n_{R}}\right)}^{2}N_{0}}\right)}^{i}}{i!})\\ \;\times [\frac{{\left(t-d_{2}\right)}^{n_{T}+n_{R}-2}}{\Gamma (n_{T}+n_{R}-1)}\text{exp}(-t+d_{2})]dt\\ \;=\sum_{i=0}^{l+nr-2}\frac{\text{exp}(\frac{d_{1}}{N_{0}}+d_{2})}{\Gamma (n_{T}+n_{R}-1)}{\left(\frac{1}{{\left(1+\sqrt{n_{R}}\right)}^{2}N_{0}}\right)}^{i}\frac{1}{i!}\int_{d_{2}}^{\infty }\text{exp}(-\frac{E_{s}ct}{{\left(1+\sqrt{n_{R}}\right)}^{2}N_{0}})\\ \;\times {\left(E_{s}ct-{\left(1+\sqrt{n_{R}}\right)}^{2}d_{1}\right)}^{i}{\left(t-d_{2}\right)}^{n_{T}+n_{R}-2}\text{exp}(-t)dt. \end{array}$$

From Proposition 5.1,

$$\begin{array}{c} \int_{d_{2}}^{\infty }(\frac{\Gamma (l+n_{R}-1,\frac{E_{s}ct-{\left(1+\sqrt{n_{R}}\right)}^{2}d_{1}}{{\left(1+\sqrt{n_{R}}\right)}^{2}N_{0}})}{\Gamma (l+n_{R}-1)})[\frac{{\left(t-d_{2}\right)}^{n_{T}+n_{R}-2}}{\Gamma (n_{T}+n_{R}-1)}\text{exp}\left(-t+d_{2}\right)]dt\\ =\sum_{i=0}^{l+nr-2}\{(\frac{\text{exp}(\frac{d_{1}}{N_{0}}+d_{2})}{\Gamma (n_{T}+n_{R}-1)}{\left(\frac{1}{{\left(1+\sqrt{n_{R}}\right)}^{2}N_{0}}\right)}^{i}\frac{1}{i!})\sum_{j=0}^{i}({\left(-1\right)}^{j}(\begin{array}{c} i\\ j \end{array})\\ {\left(E_{s}c\right)}^{i-j}{\left({\left(1+\sqrt{n_{R}}\right)}^{2}d_{1}\right)}^{j}{\left(\frac{{\left(1+\sqrt{n_{R}}\right)}^{2}N_{0}+E_{s}c}{{\left(1+\sqrt{n_{R}}\right)}^{2}N_{0}}\right)}^{j-i-n_{T}-n_{R}+1}\\ \times \Gamma \left(-j+i+n_{T}+n_{R}-1\right)\text{exp}(-\frac{d_{2}}{{\left(1+\sqrt{n_{R}}\right)}^{2}N_{0}}\left(E_{s}c+{\left(1+\sqrt{n_{R}}\right)}^{2}N_{0}\right))\\ \times 1F1[j-i,j-i-n_{T}-n_{R}+1,\frac{d_{2}}{{\left(1+\sqrt{n_{R}}\right)}^{2}N_{0}}\left(E_{s}c+{\left(1+\sqrt{n_{R}}\right)}^{2}N_{0}\right)]\}\\ =\text{exp}(\frac{-E_{s}cd_{2}+{\left(1+\sqrt{n_{R}}\right)}^{2}d_{1}}{{\left(1+\sqrt{n_{R}}\right)}^{2}N_{0}})\sum_{i=0}^{l+nr-2}\{{\left(\frac{1}{{\left(1+\sqrt{n_{R}}\right)}^{2}N_{0}}\right)}^{i}\frac{1}{i!}\sum_{j=0}^{i}{\left(-1\right)}^{j}(\begin{array}{c} i\\ j \end{array})\\ \times {\left(E_{s}c\right)}^{i-j}{\left({\left(1+\sqrt{n_{R}}\right)}^{2}d_{1}\right)}^{j}{\left(\frac{{\left(1+\sqrt{n_{R}}\right)}^{2}N_{0}+E_{s}c}{{\left(1+\sqrt{n_{R}}\right)}^{2}N_{0}}\right)}^{j-i-n_{T}-n_{R}+1}\\ \times \frac{\Gamma (-j+i+n_{T}+n_{R}-1)}{\Gamma (n_{T}+n_{R}-1)}\\ \times 1F1[j-i,j-i-n_{T}-n_{R}+2,\frac{d_{2}}{{\left(1+\sqrt{n_{R}}\right)}^{2}N_{0}}\left(E_{s}c+{\left(1+\sqrt{n_{R}}\right)}^{2}N_{0}\right)]\}\\ =\text{exp}(\frac{-E_{s}cd_{2}+{\left(1+\sqrt{n_{R}}\right)}^{2}d_{1}}{{\left(1+\sqrt{n_{R}}\right)}^{2}N_{0}})\sum_{i=0}^{l+nr-2}\{{\left(\frac{1}{{\left(1+\sqrt{n_{R}}\right)}^{2}N_{0}}\right)}^{i}\frac{1}{i!}\sum_{j=0}^{i}{\left(-1\right)}^{j}(\begin{array}{c} i\\ j \end{array})\\ \times {\left(E_{s}c\right)}^{i-j}{\left(\frac{{\left(1+\sqrt{n_{R}}\right)}^{2}N_{0}+E_{s}c}{{\left(1+\sqrt{n_{R}}\right)}^{2}N_{0}}\right)}^{j-i-n_{T}-n_{R}+1}{\left(n_{T}+n_{R}-1\right)}_{i-j}\\ \times {\left({\left(1+\sqrt{n_{R}}\right)}^{2}d_{1}\right)}^{j}1F1[j-i,j-i-n_{T}-n_{R}+2,\frac{d_{2}}{{\left(1+\sqrt{n_{R}}\right)}^{2}N_{0}}\left(E_{s}c+{\left(1+\sqrt{n_{R}}\right)}^{2}N_{0}\right)]\}\\ =\text{exp}(\frac{-E_{s}cd_{2}+{\left(1+\sqrt{n_{R}}\right)}^{2}d_{1}}{{\left(1+\sqrt{n_{R}}\right)}^{2}N_{0}})\sum_{i=0}^{l+nr-2}\{{\left(\frac{1}{{\left(1+\sqrt{n_{R}}\right)}^{2}N_{0}}\right)}^{i}\frac{1}{i!}\sum_{j=0}^{i}{\left(-1\right)}^{j}(\begin{array}{c} i\\ j \end{array})\\ \times {\left(E_{s}c\right)}^{i-j}{\left({\left(1+\sqrt{n_{R}}\right)}^{2}d_{1}\right)}^{j}{\left(\frac{{\left(1+\sqrt{n_{R}}\right)}^{2}N_{0}+E_{s}c}{{\left(1+\sqrt{n_{R}}\right)}^{2}N_{0}}\right)}^{j-i-n_{T}-n_{R}+1}{\left(n_{T}+n_{R}-1\right)}_{i-j}\\ \times (\sum_{k=0}^{i-j}\frac{{\left(j-i\right)}_{k}}{{\left(j-i-n_{T}-n_{R}+2\right)}_{k}}{\left(\frac{d_{2}}{{\left(1+\sqrt{n_{R}}\right)}^{2}N_{0}}\left(E_{s}c+{\left(1+\sqrt{n_{R}}\right)}^{2}N_{0}\right)\right)}^{k}\frac{1}{k!})\}\\ =\text{exp}(\frac{-E_{s}cd_{2}+{\left(1+\sqrt{n_{R}}\right)}^{2}d_{1}}{{\left(1+\sqrt{n_{R}}\right)}^{2}N_{0}})\sum_{i=0}^{l+nr-2}\sum_{j=0}^{i}\sum_{k=0}^{i-j}\{{\left(\frac{1}{{\left(1+\sqrt{n_{R}}\right)}^{2}N_{0}}\right)}^{i}\frac{1}{i!}{\left(-1\right)}^{j}\\ \times (\begin{array}{c} i\\ j \end{array}){\left(E_{s}c\right)}^{i-j}{\left({\left(1+\sqrt{n_{R}}\right)}^{2}d_{1}\right)}^{j}{\left(\frac{{\left(1+\sqrt{n_{R}}\right)}^{2}N_{0}+c}{{\left(1+\sqrt{n_{R}}\right)}^{2}N_{0}}\right)}^{j-i-n_{T}-n_{R}+1}\\ \times {\left(n_{T}+n_{R}-1\right)}_{i-j}(\frac{{\left(j-i\right)}_{k}}{{\left(j-i-n_{T}-n_{R}+2\right)}_{k}}\\ \times {\left(\frac{d_{2}}{{\left(1+\sqrt{n_{R}}\right)}^{2}N_{0}}\left(E_{s}c+{\left(1+\sqrt{n_{R}}\right)}^{2}N_{0}\right)\right)}^{k}\frac{1}{k!})\}\\ =\sum_{i=0}^{l+nr-2}\sum_{j=0}^{i}\sum_{k=0}^{i-j}\{{\left(\frac{1}{{\left(1+\sqrt{n_{R}}\right)}^{2}N_{0}}\right)}^{j+k-n_{T}-n_{R}+1}\frac{{\left(-1\right)}^{j}}{i!\cdot k!}(\begin{array}{c} i\\ j \end{array})\\ \times {\left({\left(1+\sqrt{n_{R}}\right)}^{2}d_{1}\right)}^{j}d_{2}^{k}{\left(n_{T}+n_{R}-1\right)}_{i-j}\frac{{\left(j-i\right)}_{k}}{{\left(j-i-n_{T}-n_{R}+2\right)}_{k}}\\ \times {\left(E_{s}c\right)}^{i-j}{\left({\left(1+\sqrt{n_{R}}\right)}^{2}N_{0}+E_{s}c\right)}^{j-i+k-n_{T}-n_{R}+1}\\ \times \text{exp}(\frac{-E_{s}cd_{2}+{\left(1+\sqrt{n_{R}}\right)}^{2}d_{1}}{{\left(1+\sqrt{n_{R}}\right)}^{2}N_{0}})\}\;, \end{array}$$

and the result follows.

Theorem 5.2 presents an approximation for the error probability of the sent codeword *C* wrongly decoded by *E*, in a transmission on a MIMO channel with a quasi-static coherent flat Rayleigh fading, where the maximum likelihood decoder is endowed with spectral norm. Thus, to obtain STBC with small error probability, we need to find codes which minimize the expression in Theorem 5.2. For fixed *E*_*s*_, *n*_*T*_, *n*_*R*_, *l* and *N*_0_, (14) is a decreasing function of *c*. In short.

(**Largest Eigenvalue Criterion**) To design a space-time block code in a MIMO communication channel with slow Rayleigh fading, we need to determine a finite family of matrices C⊂M, such that

$$\text{min}\{∥A-B{∥}_{2}:A,B\in C\;\text{and}\;A\neq B\}$$ (17)

is as large as possible.

Using the largest eigenvalue criterion, we obtain an upper bound for the pairwise error probability (PEP). Suppose a codeword *C* was sent and incorrectly decoded as *E* ≠ *C*. In this case, if the SNR is finite, the PEP is bounded by [51]

$$P(C\to E)\leq {\left[1+\gamma_{d}\;trace\left(\Delta \Delta^{*}\right)\right]}^{-n_{R}},$$

where Δ = *C* − *E*, $\gamma_{d}=\frac{1}{4\sigma^{2}}$ is a constant value proportional to SNR, and *σ*^2^ is the noise variance. Since $trace\left(\Delta \Delta^{*}\right)={∥\Delta ∥}_{F}^{2}$, from Proposition 3.1, we have

$$P(C\to E)\leq [1+\gamma_{d}{∥\Delta ∥}_{2}^{2}{]}^{-n_{R}}.$$

If *μ* is the minimum given in (17), we obtain the following upper bound for the PEP of any codewords *X* ≠ *E*

$$P\left(X-E\right)\leq {\left[1+\gamma_{d}\mu^{2}\right]}^{-n_{R}}.$$ (18)

We will refer to (18) as *spectral bound*.

## 6 Performance analysis, comparisons and examples

Let *PF* be the error probability bound giving in (2), used in the Rank and Determinant Criterion, *PT* be the bound given in (3), used in the Trace Criterion and *PE* the expression (14) of Theorem 5.2. They may be seen either as a function of the variable *c* = *c*_*F*_ = ∥*E* − *C*∥_*F*_ = ∏λ, *c* = *c*_*T*_ = |*trace*(*E* − *C*)| = ∑λ and *c* = *c*_*E*_ = ∥*E* − *C*∥_2_, respectively for *PF*, *PT* and *PE*, or as functions of variable *E*_*s*_, for fixed *c* and *N*_0_.

**Fig 4:**
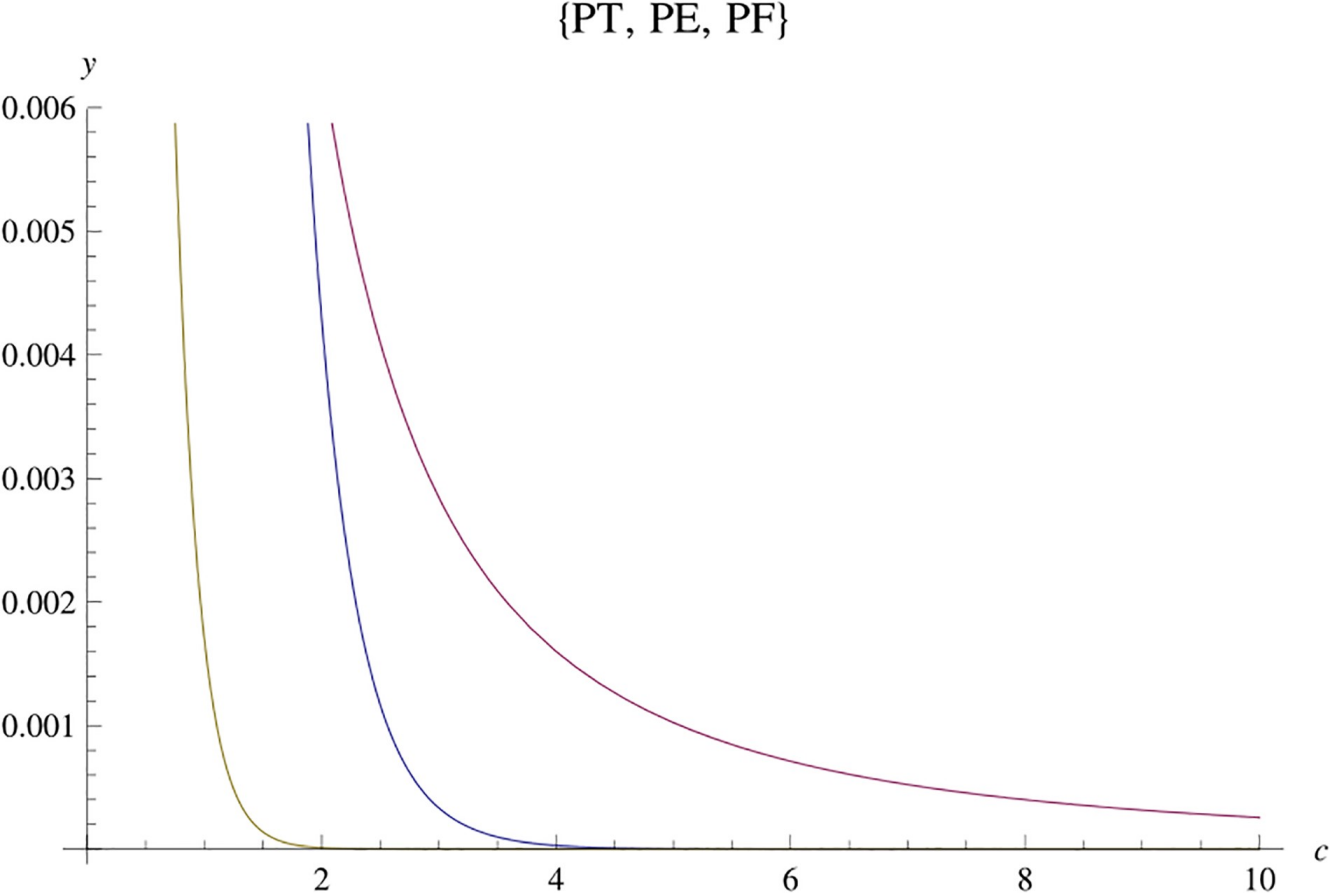
Graphs of *PT*, *PE* and *PF* for *n*_*T*_ = *n*_*R*_ = *l* = 2.

**i**) Considering *c* as variable, Fig 4 shows, from left to right, the curves of *PT*, *PE* and *PF*, where the parameters are *n*_*T*_ = *n*_*R*_ = *l* = *r* = 2, *N*_0_ = 1 and *E*_*s*_ = 10.

**Fig 5:**
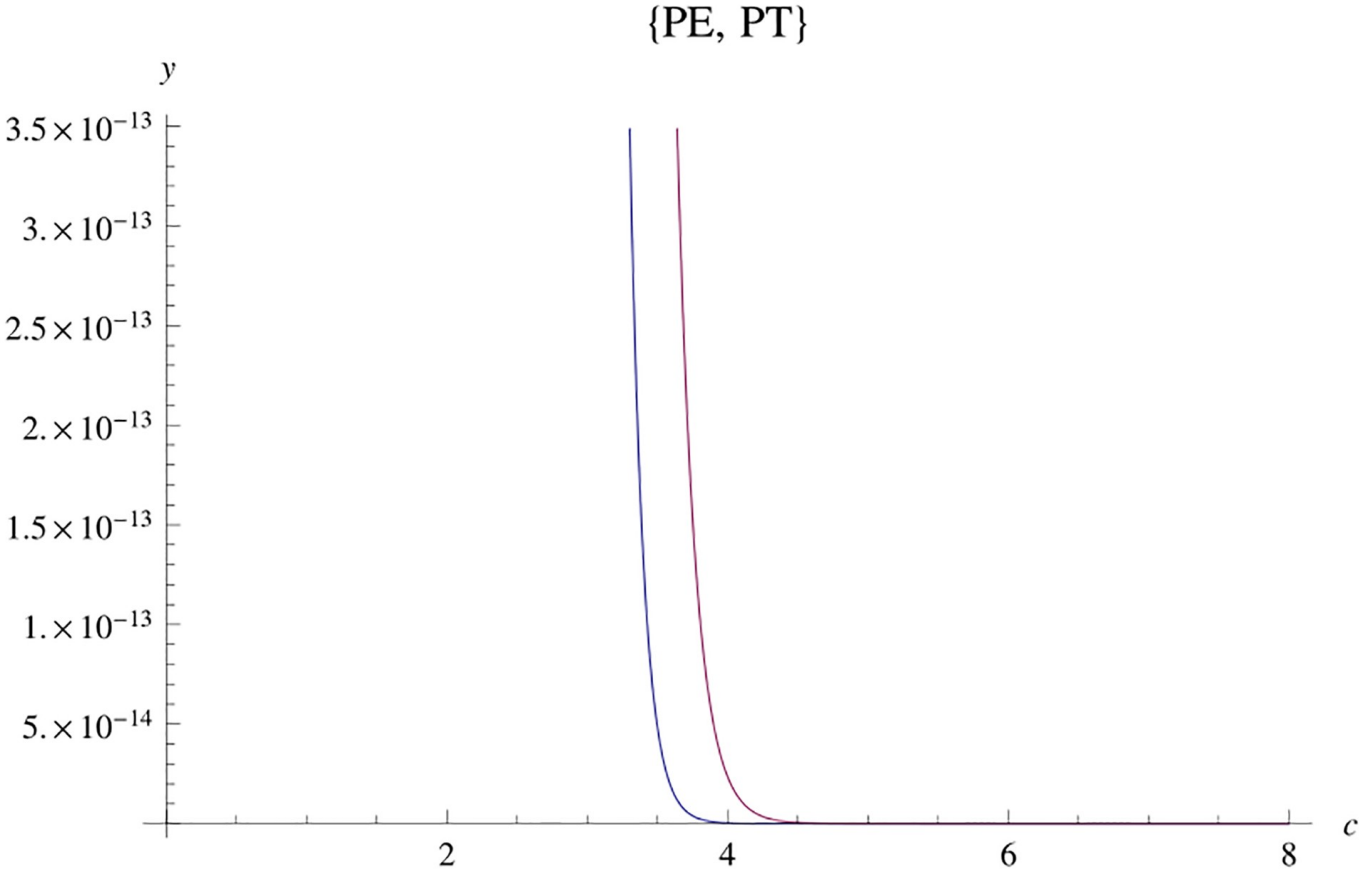
Graphs of *PE* and *PT* for *n*_*T*_ = *n*_*R*_ = *l* = 3.

**ii**) Fig 5 shows, from left to right, the curves of *PE* and *PT*, where the parameters are *n*_*T*_ = *n*_*R*_ = *l* = *r* = 3, *N*_0_ = 1 and *E*_*s*_ = 10.

In each case, the variable *c* is a fundamental parameter to choose good STBC for the criterion given. Figs 4 and 5 show that, if we choose a code C where *c*_*E*_ is greater or approximately equal to *c*_*F*_ and *c*_*T*_, we will have a very low error probability, and, for the largest eigenvalue criterion, we have much more freedom to choose C.

**Fig 6:**
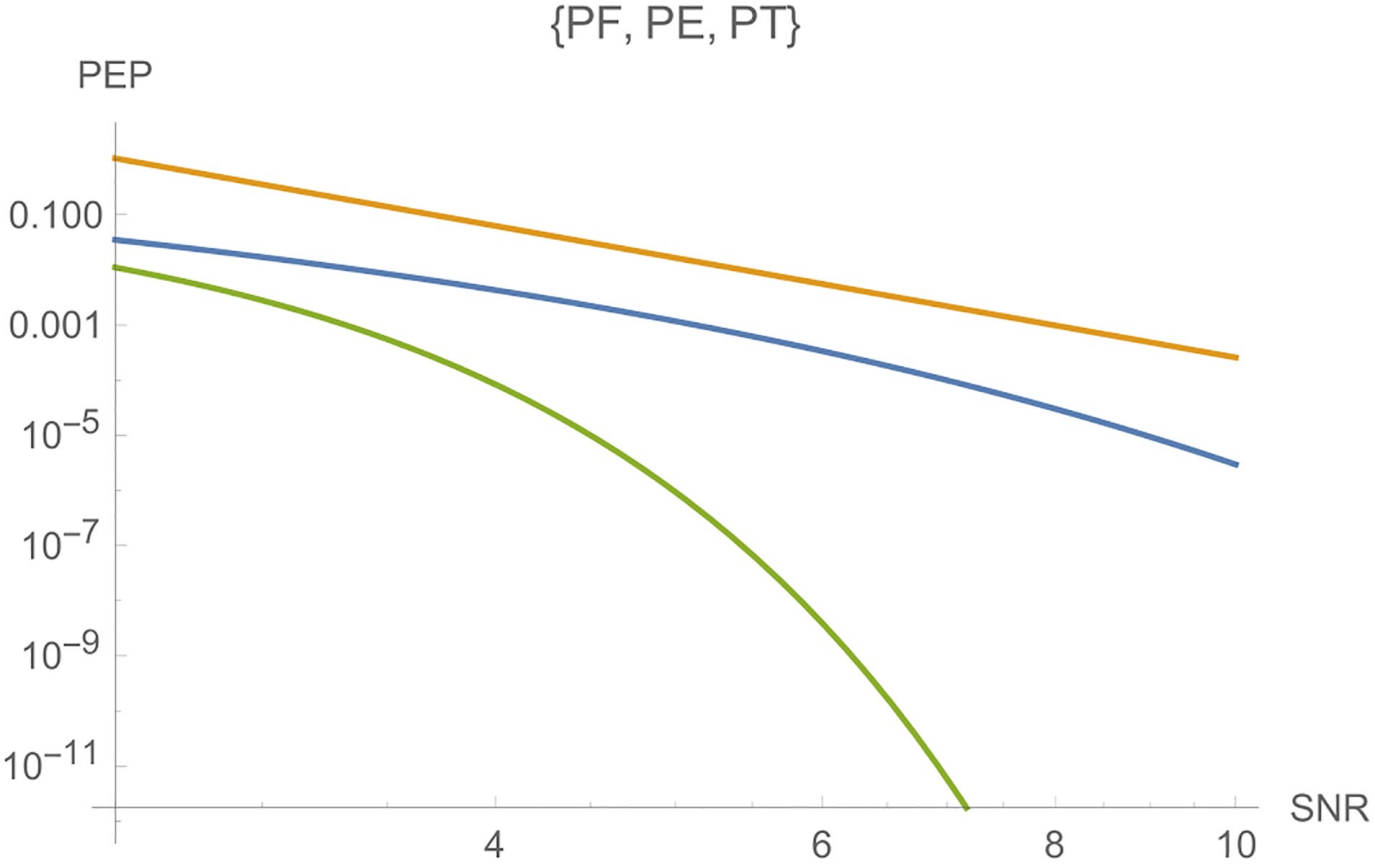
Graphs of *PT*, *PE* and *PF* for *n*_*T*_ = *n*_*R*_ = *l* = 2, where the *SNR* is a function of *E*_*s*_, for *c* = 5.

**Fig 7:**
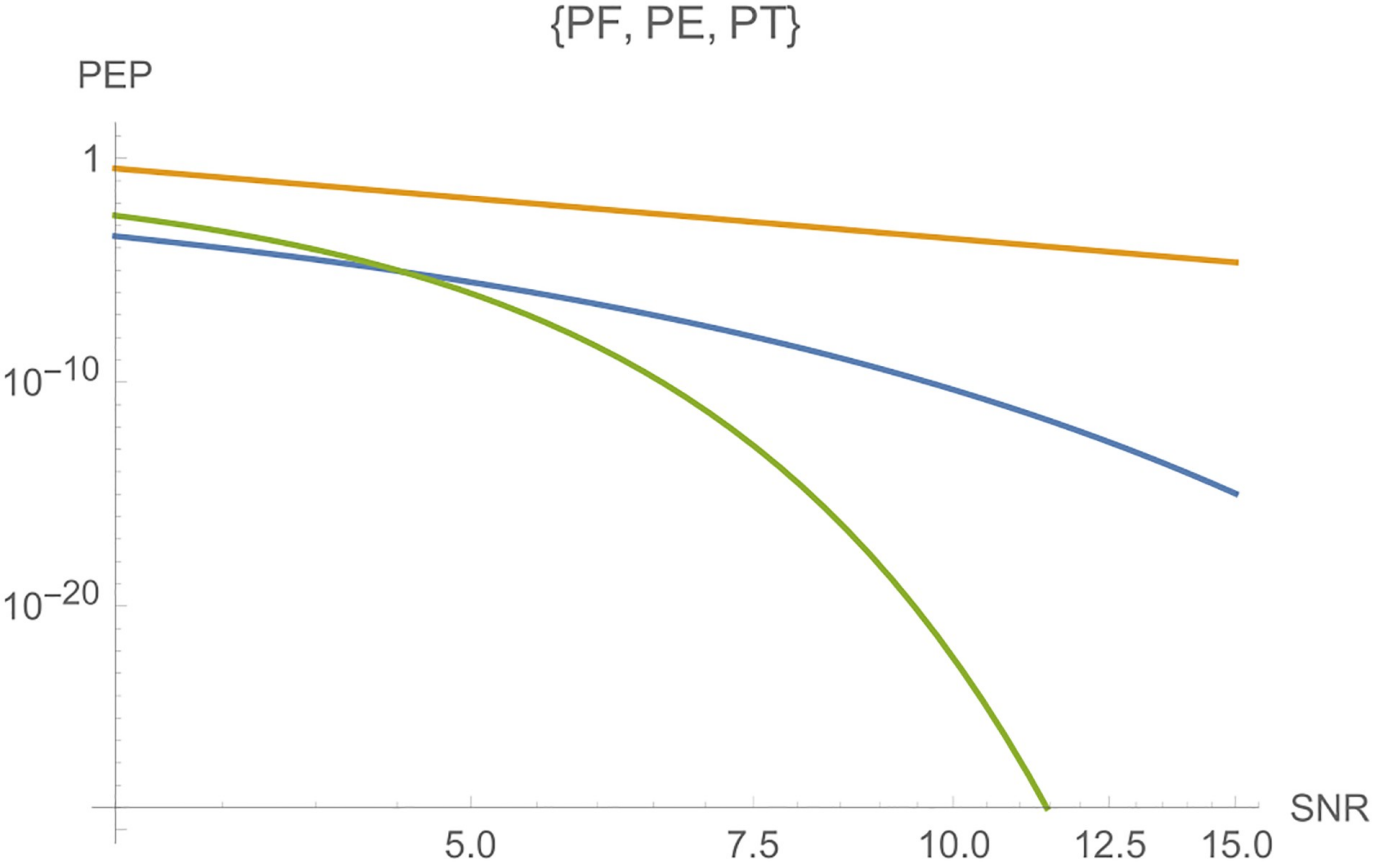
Graphs of *PE*, *PT* and *PF* for *n*_*T*_ = *n*_*R*_ = *l* = 2, where the *SNR* is a function of *E*_*s*_, for *c* = 10.

**iii**) On the other hand, Figs 6 and 7 shows, from top to botton, the curves for *PF*, *PE* and *PT*, respectively, in function of *E*_*s*_ for a fixed *c*. The parameters are *n*_*T*_ = *n*_*R*_ = *l* = *r* = 2, *N*_0_ = 1 and *c* = 5 in Fig 6. In Fig 7, we have the same parameters, where *c* = 10.

### 6.1 Examples of STBC

Now we will consider several examples of STBC and their pairwise error probabilities will be calculated.

**Example 1**. The Alamouti SBTC A for BPSK {−1, 1} constellation is given by

$$\begin{array}{c} \left\{ (\begin{array}{cc} 1 & -1\\ 1 & 1 \end{array}),(\begin{array}{cc} -1 & 1\\ -1 & -1 \end{array}),(\begin{array}{cc} 1 & 1\\ -1 & 1 \end{array}),(\begin{array}{cc} -1 & -1\\ 1 & -1 \end{array})\}\;.\right. \end{array}$$

Let us consider also the SBTC A1 given by

$$\begin{array}{c} \left\{ (\begin{array}{cc} 1 & -1\\ -1 & 1 \end{array}),(\begin{array}{cc} -1 & 1\\ 1 & -1 \end{array}),(\begin{array}{cc} 1 & 1\\ 1 & 1 \end{array}),(\begin{array}{cc} -1 & -1\\ -1 & -1 \end{array})\}.\right. \end{array}$$

**Fig 8:**
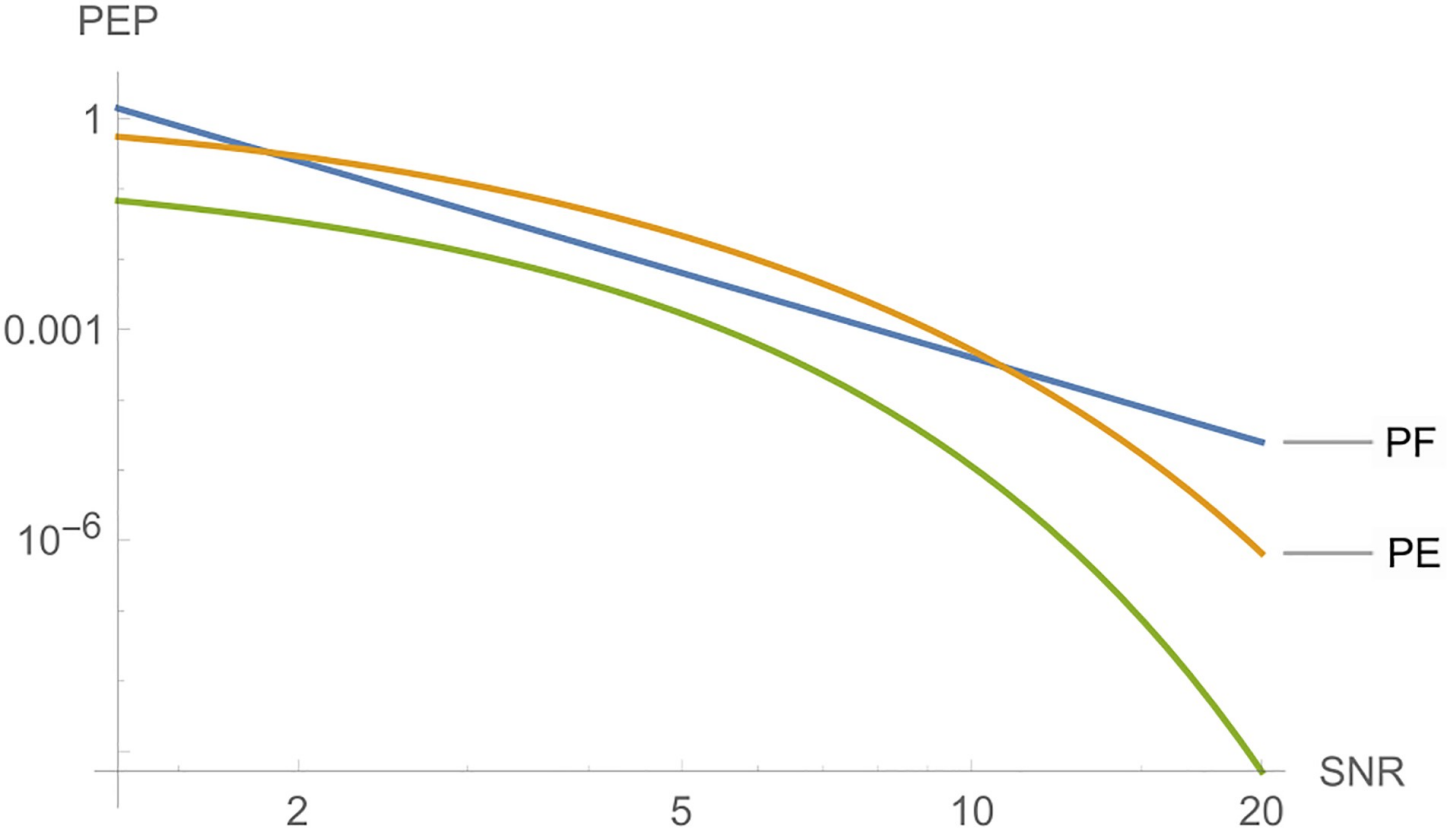
Graphs of *PE*, *PF* and *PT* for A of Example 1, where *n*_*T*_ = *n*_*R*_ = *l* = 2, where SNR is a function of *E*_*s*_.

Both SBTC have the same rate. For A, $c_{F}={\text{max}}_{A,A^{\prime }\in A}det\left(A-A^{\prime }\right)=8$, thus *PF*(8) ≈ 0.0004, $c_{E}={\text{max}}_{A,A^{\prime }\in A}{∥A-A^{\prime }∥}_{2}=2\sqrt{2}$ and $PE(2\sqrt{2})\approx 0.0005$. We also have ${\text{max}}_{A,A^{\prime }\in A}rank\left(A-A^{\prime }\right)=2$. Taking $c_{T}={\text{max}}_{A,A^{\prime }\in A}trace\left(A-A^{\prime }\right)=4$, in Fig 8, we show the *PEP* for each case, with *N*_0_ = 1.

**Fig 9:**
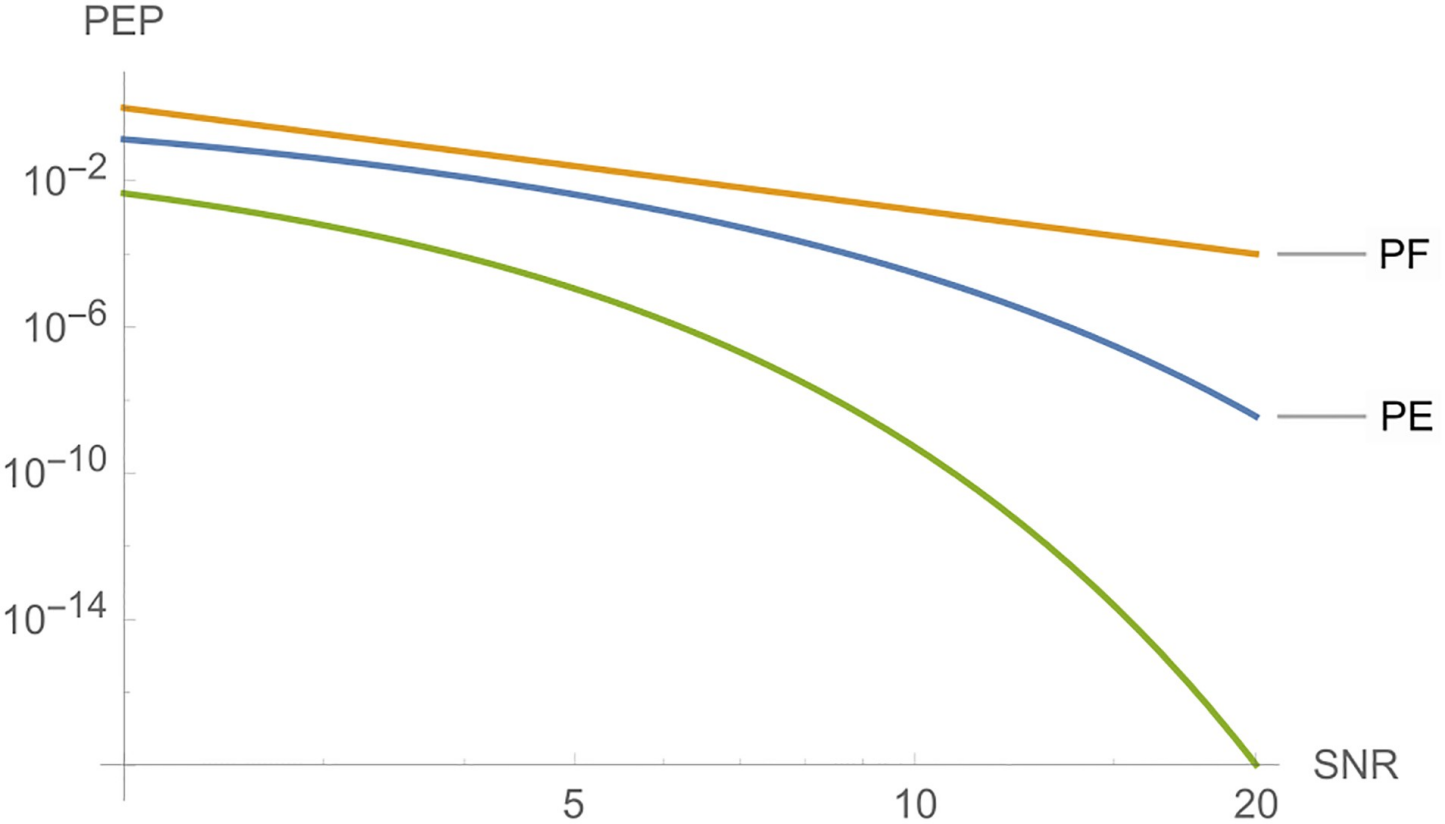
Graphs of *PE*, *PF* and *PT* for A1 of Example 1, where *n*_*T*_ = *n*_*R*_ = *l* = 2, and *SNR* is a function of *E*_*s*_.

Now, for A1 one has $c_{T}={\text{max}}_{B,B^{\prime }\in A1}{∥B-B^{\prime }∥}_{2}=4$ and *PE*(4) ≈ 0.00002. On the other hand, $c_{T}={\text{max}}_{B,B^{\prime }\in A1}trace\left(B-B\right)=2$ and $c_{F}={\text{max}}_{B,B^{\prime }\in A1}det\left(B-B^{\prime }\right)=4$, thus, *PF*(4) ≈ 0.0016. Therefore, for the Alamouti code A, one has similar performances. However, for STBC A1 the performance of spectral case is better than the Rank-Determinant case. Values are shown in Table 4. In Fig 9, we show the *PEP* for each case. Since this SBTC does not have full diversity, it would not be used by the two classical criteria. Assuming spectral norm, it can be used.

**Table 4:** Comparisons between the two STC.

-	A	A1
PF	0.0004	0.0016
PE	0.0005	0.00002

**Example 2**. Considering again the Alamouti STBC A, albeit for QPSK $\left\{\frac{1}{\sqrt{2}}\left(1+j\right),\frac{1}{\sqrt{2}}\left(-1+j\right),\frac{1}{\sqrt{2}}\left(-1-j\right),\frac{1}{\sqrt{2}}\left(1-j\right)\right\}$ constellation. We have a code with 16 matrices.

**Fig 10:**
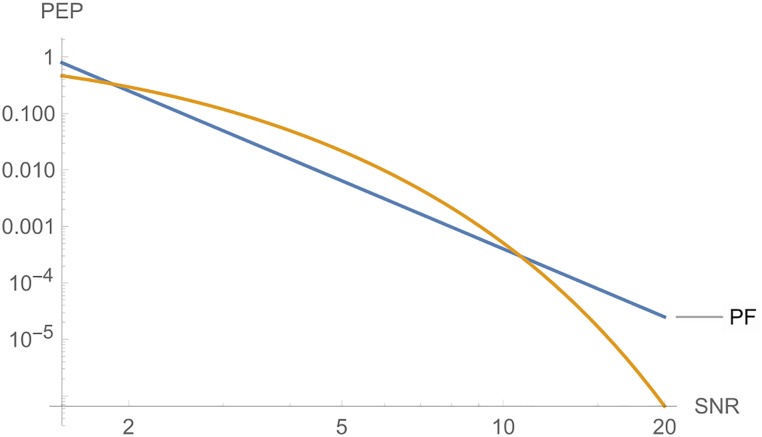
Graphs of *PE*, *PF* and *PT* for Example 2, where *n*_*T*_ = *n*_*R*_ = *l* = 2, and *SNR* is a function of *E*_*s*_.

We have that ${\text{max}}_{A,A^{\prime }\in A}det\left(A-A^{\prime }\right)=8$, thus *PF*(8) ≈ 0.0004, but ${\text{max}}_{A,A^{\prime }\in A}{∥A-A^{\prime }∥}_{2}=2\sqrt{2}$ and $PE(2\sqrt{2})\approx 0.0005$. Fig 10 shows the *PEP* for the two cases, for *N*_0_ = 1. We see that, for a *SNR* > 10, the *PEP* for the spectral case is much lower than the rank and determinant case.

**Example 3**. Jafarkhani proved that we have an orthogonal STBC only for two, four and eight antennas. Let C be the orthogonal STBC for four antennas, where *n*_*T*_ = *n*_*R*_ = *l* = 4, considering the BPSK {−1, 1} constellation. The 16 matrices of C are in the form

$$\begin{array}{c} C=(\begin{array}{cccc} x_{1} & x_{2} & x_{3} & x_{4}\\ -x_{2} & x_{1} & -x_{4} & x_{3}\\ -x_{3} & x_{4} & x_{1} & -x_{2}\\ -x_{4} & -x_{3} & x_{2} & x_{1} \end{array})\;. \end{array}$$

**Fig 11:**
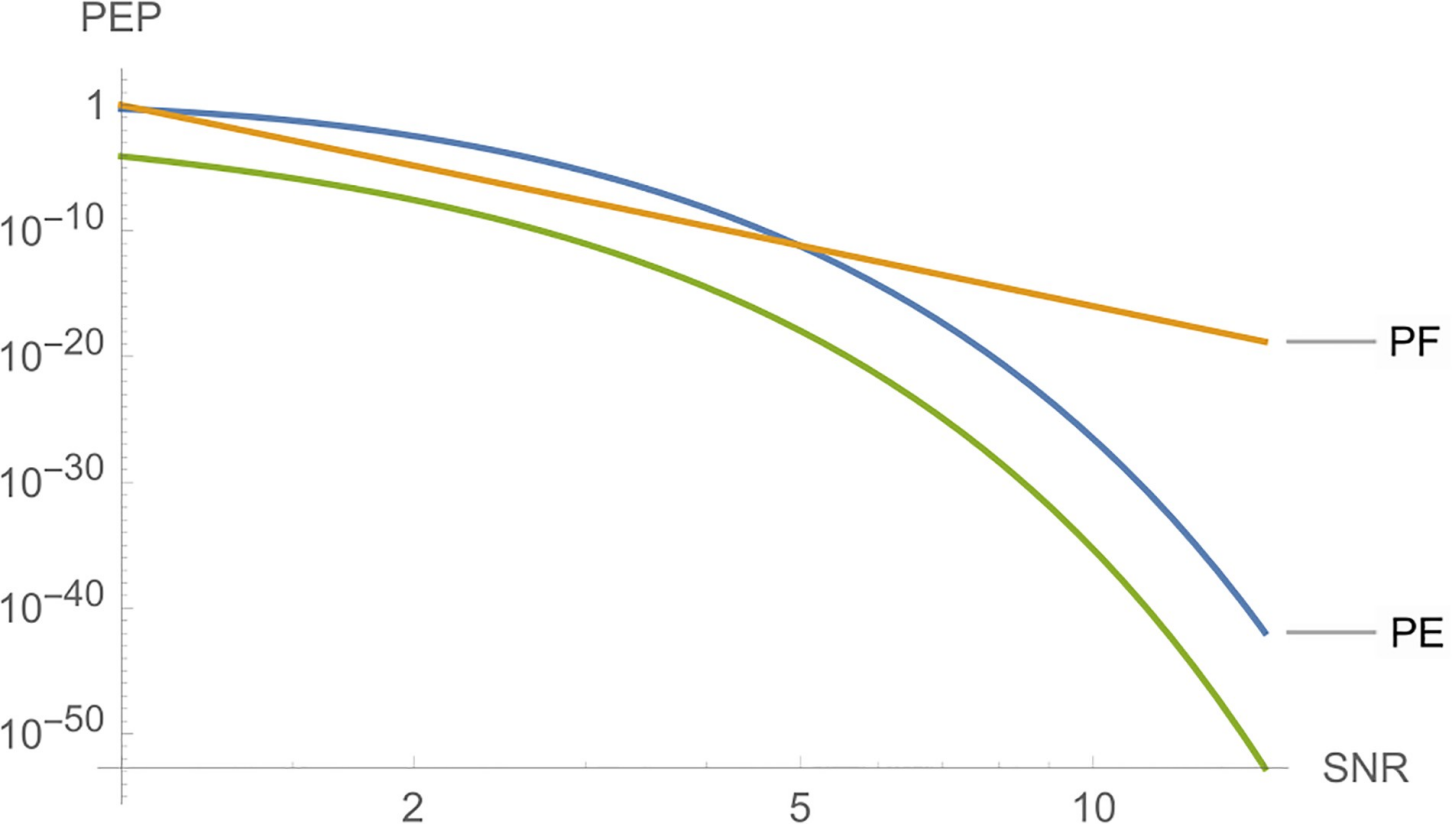
Graphs of *PE*, *PF* and *PT* for Example 3, where *n*_*T*_ = *n*_*R*_ = *l* = 4, and *SNR* is a function of *E*_*s*_.

With regard to this code, we have $c_{F}={\text{max}}_{A,A^{\prime }\in C}\left|det\left(A-A^{\prime }\right)\right|=256$, $c_{E}={\text{max}}_{A,A^{\prime }\in C}{∥A-A^{\prime }∥}_{2}=4$ and $c_{T}={\text{max}}_{A,A^{\prime }\in C}\left|trace\left(A-A^{\prime }\right)\right|=8$. With these parameters, Fig 11 compares the three cases, in which we have a fast decrease of *PEP* for *PE* and *PT*.

**Example 4**. In [52], Jafarkhani introduced the Quasi-orthogonal STBC. In the case of four antennas, we have the code C, where *n*_*T*_ = *n*_*R*_ = *l* = 4, considering the BPSK {−1, 1} constellation. The 16 matrices of C are in the form

$$\begin{array}{c} C=(\begin{array}{cccc} x_{1} & x_{2} & x_{3} & x_{4}\\ -x_{2} & x_{1} & -x_{4} & x_{3}\\ -x_{3} & -x_{4} & x_{1} & x_{2}\\ x_{4} & -x_{3} & -x_{2} & x_{1} \end{array})\;. \end{array}$$

**Fig 12:**
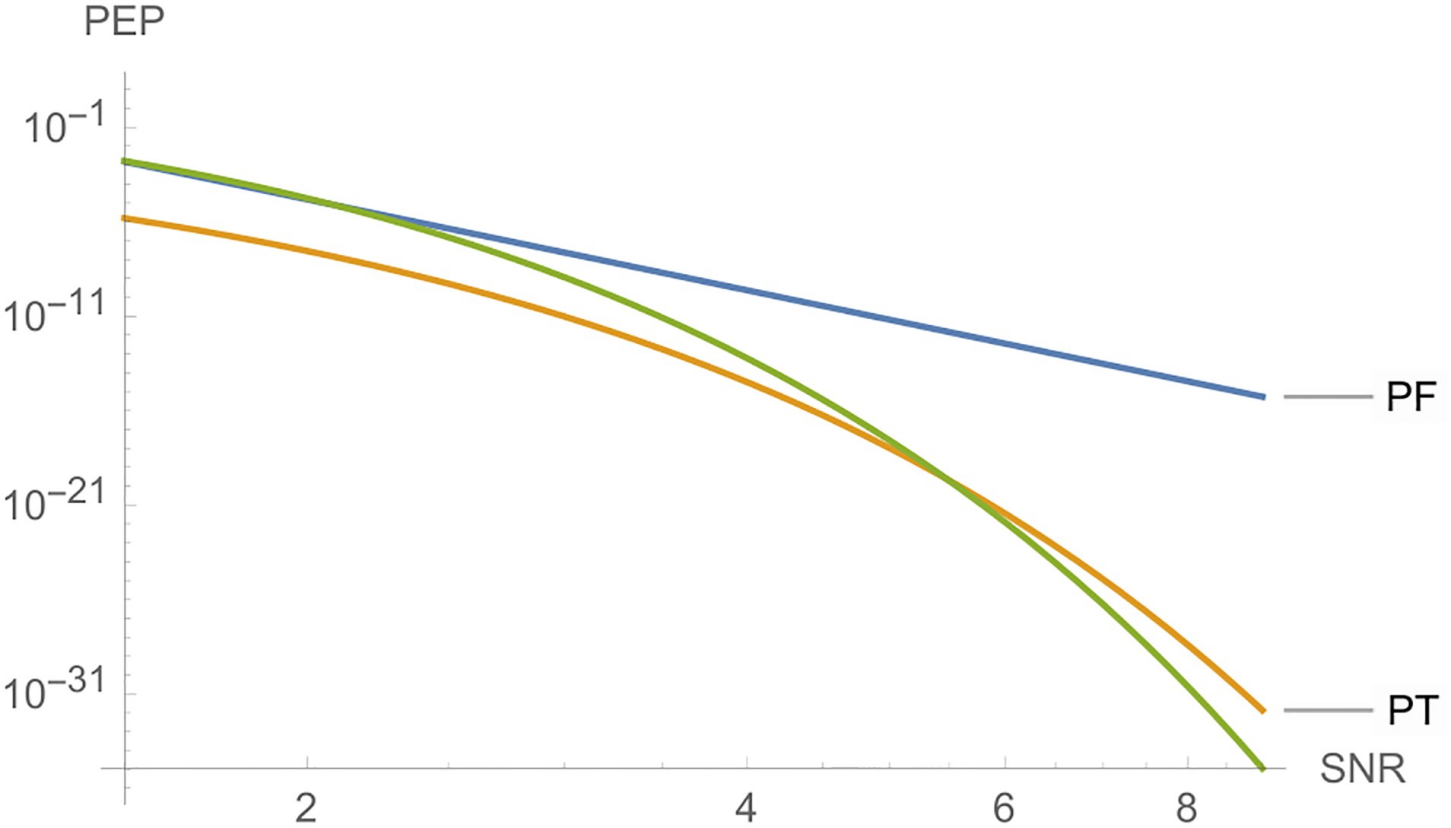
Graphs of *PE*, *PF* and *PT* for Example 4, where *n*_*T*_ = *n*_*R*_ = *l* = 4, and *SNR* is a function of *E*_*s*_.

With regard to this code, we have $c_{F}={\text{max}}_{A,A^{\prime }\in C}\left|det\left(A-A^{\prime }\right)\right|=256$, $c_{E}={\text{max}}_{A,A^{\prime }\in C}{∥A-A^{\prime }∥}_{2}=5.6568542$ and $c_{T}={\text{max}}_{A,A^{\prime }\in C}\left|trace\left(A-A^{\prime }\right)\right|=8$. With these parameters, Fig 12 compares the three cases, in which we have a fast decrease of *PEP* for *PE* and *PT*. In Example 3, we have the same values to *c*_*F*_ and *c*_*T*_, and a similar value to *c*_*E*_, albeit now we have a better *PEP* to *PE*.

The largest eigenvalue criterion may be used to choose a STBC from a given set of matrices, which usually would not be chosen by the other criteria. We can see this in the next examples.

**Example 5**. Let C be the set of matrices for BPSK {−1, 1} constellation given by the matrices

$$\begin{array}{c} \begin{array}{c} \left\{ (\begin{array}{ccc} 1 & -1 & 0\\ 1 & 1 & 0\\ 0 & 0 & 1 \end{array}),(\begin{array}{ccc} -1 & 1 & 0\\ -1 & -1 & 0\\ 0 & 0 & 1 \end{array}),(\begin{array}{ccc} 1 & 1 & 0\\ -1 & 1 & 0\\ 0 & 0 & 1 \end{array}),(\begin{array}{ccc} -1 & -1 & 0\\ 1 & -1 & 0\\ 0 & 0 & 1 \end{array}),(\begin{array}{ccc} 1 & 0 & 0\\ 0 & 1 & -1\\ 0 & 1 & 1 \end{array}),(\begin{array}{ccc} 1 & 0 & 0\\ 0 & -1 & 1\\ 0 & -1 & -1 \end{array}),\right. \end{array}\\ \begin{array}{c} (\begin{array}{ccc} 1 & 0 & 0\\ 0 & 1 & 1\\ 0 & -1 & 1 \end{array}),(\begin{array}{ccc} 1 & 0 & 0\\ 0 & -1 & -1\\ 0 & 1 & -1 \end{array}),(\begin{array}{ccc} 1 & 0 & -1\\ 0 & 1 & 0\\ 1 & 0 & 1 \end{array}),(\begin{array}{ccc} -1 & 0 & 1\\ 0 & 1 & 0\\ -1 & 0 & -1 \end{array}),(\begin{array}{ccc} 1 & 0 & 1\\ 0 & 1 & 0\\ -1 & 0 & 1 \end{array}),(\begin{array}{ccc} -1 & 0 & -1\\ 0 & 1 & 0\\ 1 & 0 & -1 \end{array})\}, \end{array} \end{array}$$

**Fig 13:**
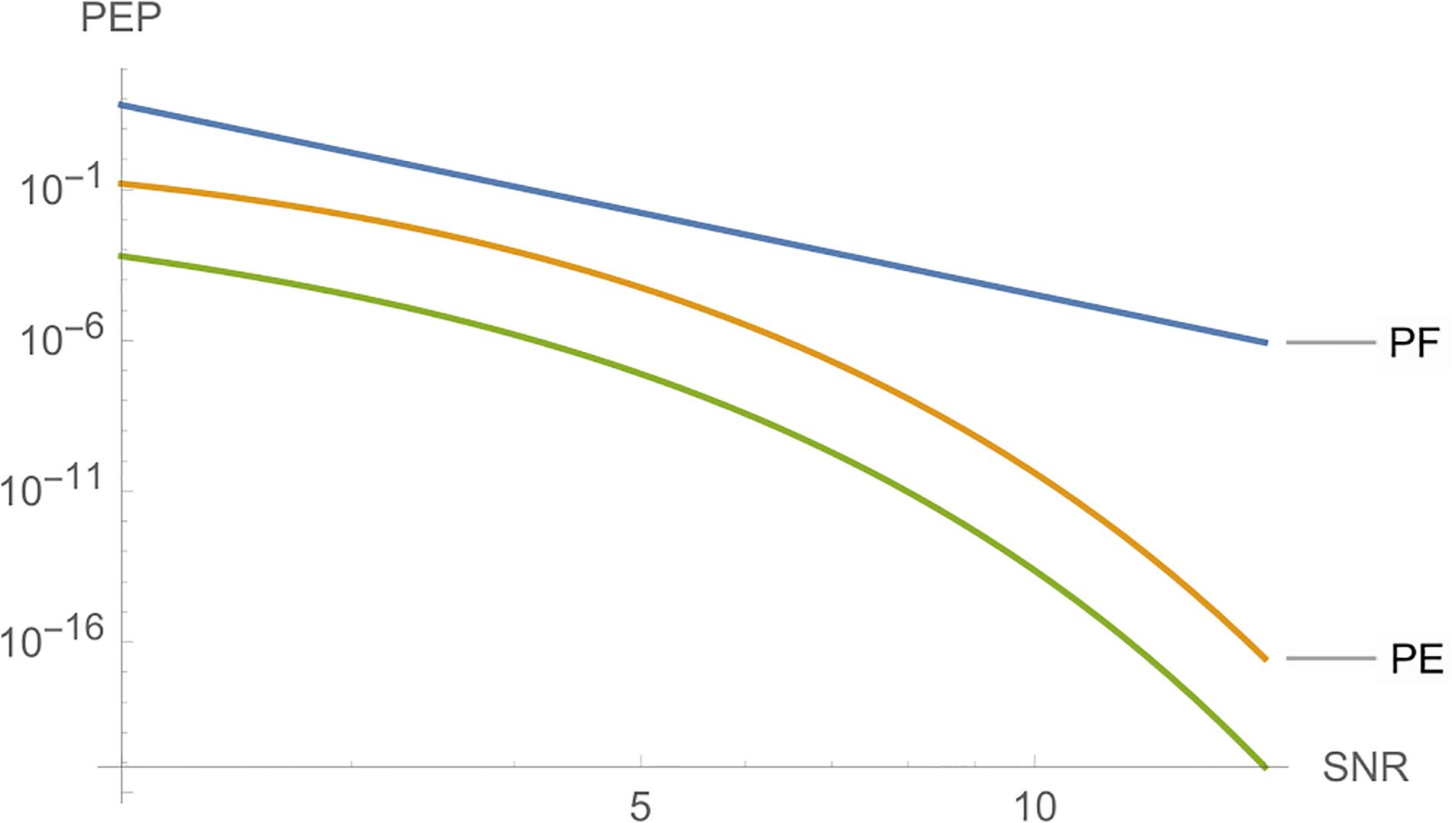
Graphs of *PE*, *PF* and *PT* for Example 5, where *n*_*T*_ = *n*_*R*_ = *l* = 3, and *SNR* is a function of *E*_*s*_.

In this set, we have 12 matrices, where the columns of each one of them is a orthogonal set of vectors. Even though we have neither an orthogonal STBC, nor a full diversity code, the set has several important properties, since it has similar mathematical properties to the Alamouti code of Example 1. It can be used for three antennas, with *n*_*T*_ = *n*_*R*_ = *l* = 3, considering the largest eingenvalue criterion. Fig 13 shows *PEP* for the three cases, where, for this code, we have $c_{F}={\text{max}}_{A,A^{\prime }\in C}\left|det\left(A-A^{\prime }\right)\right|=2$, $c_{E}={\text{max}}_{A,A^{\prime }\in C}{∥A-A^{\prime }∥}_{2}=2.828427$ and $c_{T}={\text{max}}_{A,A^{\prime }\in C}\left|trace\left(A-A^{\prime }\right)\right|=4$.

**Fig 14:**
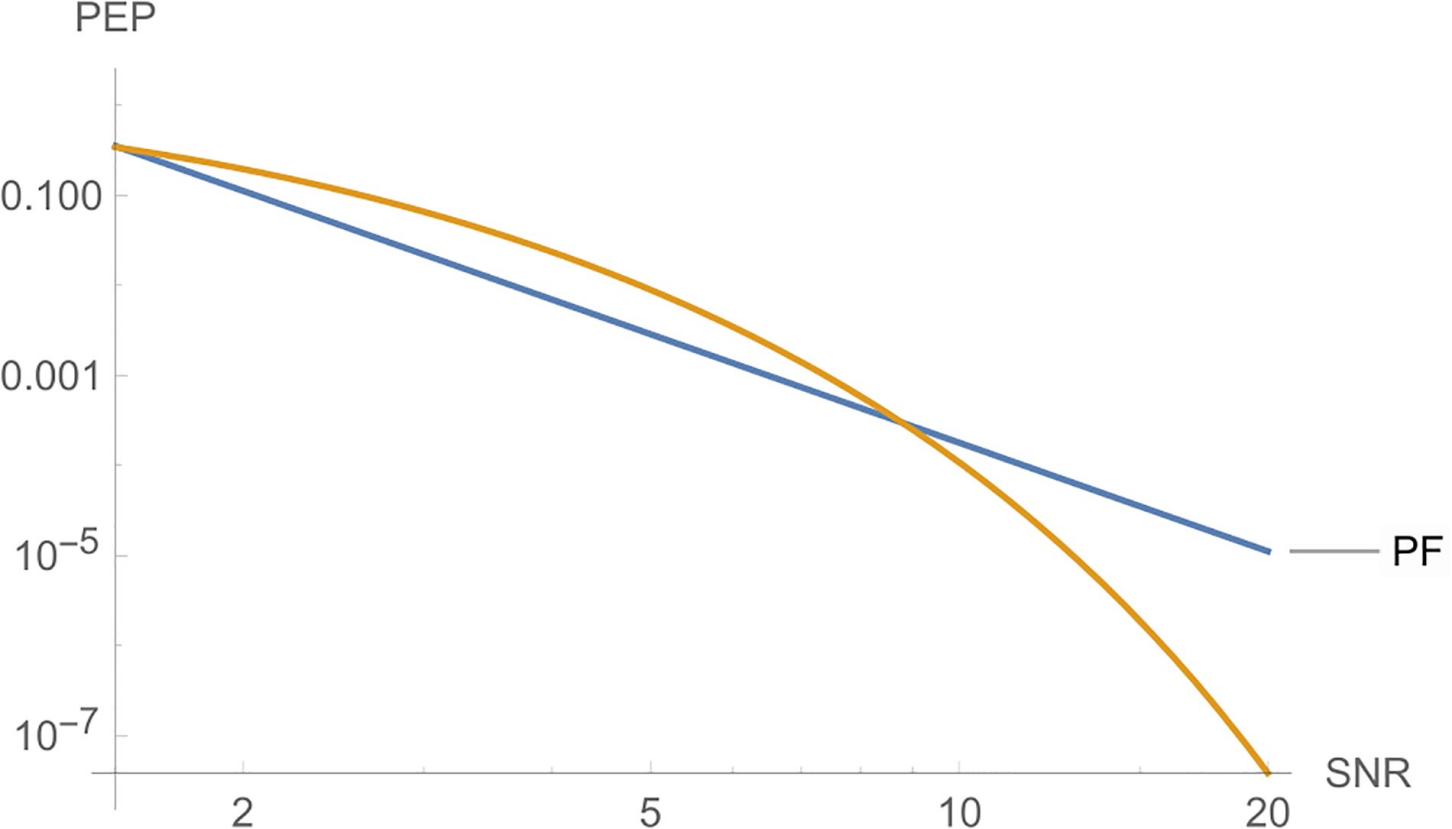
Graphs of *PE* and *PF* for Example 6, where *n*_*T*_ = *n*_*R*_ = *l* = 2, and *SNR* is a function of *E*_*s*_.

**Example 6**. Let $C=(\begin{array}{cc} xi & -\bar{z}\\ z & -xi \end{array})$, where x∈R and z∈C. We can prove that for any codeword *X* of C, ${∥X∥}_{2}=\sqrt{x^{2}+{\left|z\right|}^{2}}$. For convenient choices of the constellation, we may consider finite families of matrices where, for the differences Δ = *X* − *E*, ∥Δ∥_2_ will be as large as desired. On the other hand, since *trace*(Δ) = 0, then C cannot be used as a STBC, according to trace criterion. For instance, in C, let *z* = *a* + *ib*. For *x*, *a*, *b* ∈ {−1, 1} we have eight rank 2 matrices giving a BPSK code. Fig 14 shows *PEP* for the two cases. For this code, $c_{F}={\text{max}}_{A,A^{\prime }\in C}\left|det\left(A-A^{\prime }\right)\right|=12$ and $c_{E}={\text{max}}_{A,A^{\prime }\in C}{∥A-A^{\prime }∥}_{2}=3.4641$.

**Example 7**. [53] studies space-time group codes. An important example is the following.

**Fig 15:**
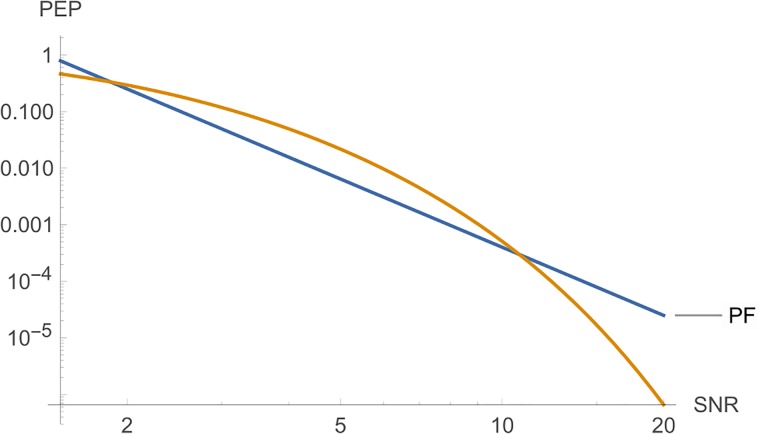
Graphs of *PE* and *PF* for Example 7, where *n*_*T*_ = *n*_*R*_ = *l* = 2, and *SNR* is a function of *E*_*s*_.

Let $G=\{\pm (\begin{array}{cc} 1 & 0\\ 0 & 1 \end{array}),\pm (\begin{array}{cc} i & 0\\ 0 & -i \end{array}),\pm (\begin{array}{cc} 0 & 1\\ -1 & 0 \end{array}),\pm (\begin{array}{cc} 0 & i\\ i & 0 \end{array})\}$ and $D=(\begin{array}{cc} 1 & -1\\ 1 & 1 \end{array})$. Then C=DG is called the Quaternion STBC, and satisfies the Rank and Determinant Criterion. One has $c_{F}={\text{max}}_{A,A^{\prime }\in C}\left|det\left(A-A^{\prime }\right)\right|=8$ and $c_{E}={\text{max}}_{A,A^{\prime }\in C}{∥A-A^{\prime }∥}_{2}=2.828427$. Fig 15 shows *PEP* for each case.

Now, if $D_{1}=(\begin{array}{cc} 1 & 1\\ 1 & 1 \end{array})$, then $C_{1}=D_{1}G$ is a new STBC with $∥C_{1}-C_{2}{∥}_{2}=4\sqrt{2}$ for all $C_{1}\neq C_{2}\in C_{1}$, but the Rank and Determinant Criterion is not satisfied.

**Example 8**. Let

$$\begin{array}{c} C=(\begin{array}{cccccccc} x_{1} & -x_{2} & -x_{3} & -x_{4} & -x_{5} & x_{6} & -x_{7} & -x_{8}\\ x_{2} & x_{1} & -x_{4} & x_{3} & -x_{6} & -x_{5} & -x_{8} & x_{7}\\ x_{3} & x_{4} & x_{1} & -x_{2} & -x_{7} & -x_{8} & x_{5} & -x_{6}\\ x_{4} & -x_{3} & x_{2} & x_{1} & -x_{8} & x_{7} & x_{6} & x_{5}\\ x_{5} & -x_{6} & x_{7} & x_{8} & x_{1} & -x_{2} & -x_{3} & -x_{4}\\ x_{6} & x_{5} & x_{8} & -x_{7} & x_{2} & x_{1} & -x_{4} & x_{3}\\ x_{7} & x_{8} & -x_{5} & x_{6} & x_{3} & x_{4} & x_{1} & -x_{2}\\ x_{8} & -x_{7} & -x_{6} & -x_{5} & x_{4} & -x_{3} & x_{2} & x_{1} \end{array})\;. \end{array}$$

This matrix appears in the study of representations of Clifford algebras. If we consider the BPSK {−1, 1} constellation, we have a code for eight antennas, with 256 matrices and *n*_*T*_ = *n*_*R*_ = *l* = 8.

**Fig 16:**
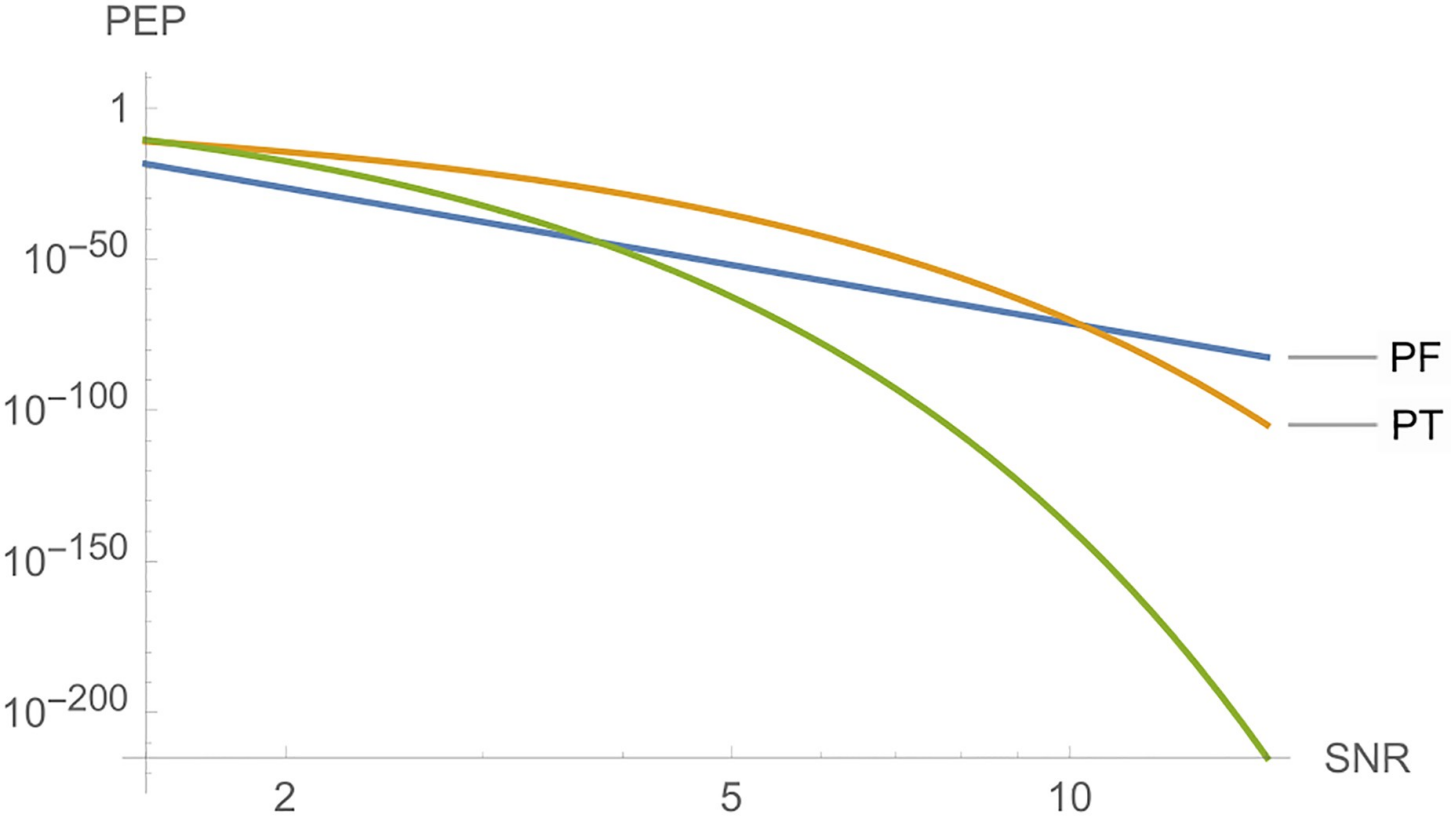
Graphs of *PF*, *PT* and *PE* for Example 8, where *n*_*T*_ = *n*_*R*_ = *l* = 8, and *SNR* is a function of *E*_*s*_.

For this code we have $c_{F}={\text{max}}_{A,A^{\prime }\in C}\left|det\left(A-A^{\prime }\right)\right|=518400$, $c_{E}={\text{max}}_{A,A^{\prime }\in C}{∥A-A^{\prime }∥}_{2}=7.2111$ and $c_{T}={\text{max}}_{A,A^{\prime }\in C}\left|trace\left(A-A^{\prime }\right)\right|=8$. With such parameters, we see that Fig 16 compares the three cases, where we have a fast decrease of *PEP* for *PE* and *PT*. Even for a really big value of *c*_*F*_, in this case, *PE* is better than the other two cases. We may also consider subsets of this code to obtain new codes.

**Remark 2**. In Figs 6 to 16, we see that, at high *SNR*, the proposed Largest Eingenvalue Criterion in better than the Rank-determinant Criterion, but it is overcome by the Trace Criterion in some cases. We recall that the Rank Criterion is obtained supposing a small *SNR*, see [46]. Thus, the comparisons at high *SNR* are theoretical. On the other hand, the proposed criterion can be used in all *SNR* regimes.

## 7 Codes from block matrices

There are several space-time block codes whose matrices are made of blocks of other matrices. In this section, we will supply some properties that will help us apply the Largest Eingenvalue Criterion for those codes.

Quasi-Orthogonal Space-Time Block Codes (QOSTBC) are a powerful family of codes used in Multiple-Input Multiple-Output (MIMO) communication systems, and they provide transmit diversity with higher code rates than the well-known orthogonal STBC (OSTBC), with a lower decoding complexity than non-orthogonal STBC. We have the BPSK case in Example 4 of last section.

We obtain results for several well-known quasi-orthogonal space-time block codes (QOSTBC) from [52] and [54]. In order to derive the main results, we need the following proposition related to the spectral norm and block matrices. Giving matrices A,B∈M=M(n,C)=M(n,n,C), by $\left(\begin{array}{cc} A & 0\\ 0 & B \end{array}\right)$, we denote the block matrix of size 2*n* × 2*n*, where 0 = 0_*n*×*n*_ ∈ M.

**Proposition 7.1** *Let A*, *B* ∈ *M*. *Then*,

(a) $∥A^{*}{A∥}_{2}=∥AA^{*}{∥}_{2}={∥A∥}_{2}^{2}\;;$
(b) $∥\left(\begin{array}{cc} A & 0\\ 0 & B \end{array}\right){∥}_{2}={\text{max}\{∥A∥}_{2}{,∥B∥}_{2}\}\;;$
(c) $∥(\begin{array}{cc} 0 & B\\ A & 0 \end{array}){∥}_{2}={\text{max}\{∥A∥}_{2}{,∥B∥}_{2}\}\;;$
(d) $∥(\begin{array}{cc} 0 & -A\\ A & 0 \end{array}){∥}_{2}={∥A∥}_{2}\;.$

**Proof**.

(a) Let ${∥A∥}_{2}^{2}=\lambda$. Then, λ is the largest eingenvalue of *A*_1_ = A*A. We have to show that λ is a singular value for both *A*_1_ = A*A and *A*_2_ = AA*. Let v≠0 be an eingenvector associated to λ. Thus, A*Av = *λv*. Applying *A*_1_ on both sides, we have:
  $$\begin{array}{cc} \left(A^{*}A\right)\left(A^{*}A\right)v & =(A^{*}A)\lambda v\Rightarrow \\ \left(A^{*}A\right)\left(A^{*}A\right)v & =\lambda (A^{*}Av)\Rightarrow \\ {\left(A^{*}A\right)}^{*}\left(A^{*}A\right)v & =\lambda^{2}v, \end{array}$$
  which means that λ^2^ is an eigenvalue for (A*A)*(A*A). Therefore, λ is a singular value for *A*_1_. The argument is similar for *A*_2_. The result then follows from the definition of the spectral norm.
(b) Suppose **x** and **y** are unit vectors with entries in C, and *a*, *b* ≥ 0 are such that *a*^2^ + *b*^2^ = 1. The vector
  $$\begin{array}{cc} v & =(\begin{array}{c} ax\\ by \end{array}) \end{array}$$
  is also a unit vector. Since every unit vector **v** can be written in this form, it follows that
  $$\begin{array}{cc} {∥(\begin{array}{cc} A & 0\\ 0 & B \end{array})v∥}_{2}^{2} & ={∥(\begin{array}{c} aAx\\ bBy \end{array})∥}_{2}^{2}\\ & =a^{2}{∥Ax∥}_{2}^{2}+b^{2}{∥By∥}_{2}^{2}\\ & \leq \left(a^{2}+b^{2}\right){[\text{max}\{∥Ax∥}_{2}{,∥By∥}_{2}{\}]}^{2}, \end{array}$$
  which concludes the affirmation.
(c) Define
  $$\begin{array}{cc} Y & =(\begin{array}{cc} 0 & B\\ A & 0 \end{array}). \end{array}$$
  From (*a*),
  $${∥Y∥}^{2}={∥(\begin{array}{cc} 0 & B\\ A & 0 \end{array})(\begin{array}{cc} 0 & A^{*}\\ B* & 0 \end{array})∥}_{2}={∥(\begin{array}{cc} BB^{*} & 0\\ 0 & AA^{*} \end{array})∥}_{2}.$$
  Using (b), it follows that
  $$\begin{array}{cc} {∥Y∥}_{2}^{2} & =\text{max}\{∥BB^{*}{∥}_{2},∥AA^{*}{∥}_{2}\}\\ & =\text{max}\{∥B{∥}_{2}^{2},∥A{∥}_{2}^{2}\}\\ & ={\left[\text{max}\{∥A{∥}_{2},∥B{∥}_{2}\}\right]}^{2}, \end{array}$$
  concluding the proof.
(d) It follows directly from (c).

Using Proposition 7.1, we find an upper bound for the spectral norm of block matrices and then use the spectral criterion to get a bound for the *PEP* for STBCs. Let Δ = *X* − *E*, where *X* ≠ *E* are codewords of a given code C. We write

$$\begin{array}{cc} \Delta & =(\begin{array}{cc} \Delta_{11} & \Delta_{12}\\ \Delta_{21} & \Delta_{22} \end{array})=(\begin{array}{cc} \Delta_{11} & 0\\ 0 & \Delta_{22} \end{array})+(\begin{array}{cc} 0 & \Delta_{12}\\ \Delta_{21} & 0 \end{array}). \end{array}$$

From (18) and Proposition 7.1, we have

$$\begin{array}{cc} P(X\to E) & \leq [1+\gamma_{d}{∥\Delta ∥}_{2}^{2}{]}^{-n_{R}}\\ & \leq [1+\gamma_{d}(\text{max}\{∥\Delta_{11}{∥}_{2},∥\Delta_{22}{∥}_{2}\}+\text{max}\{∥\Delta_{12}{∥}_{2},∥\Delta_{21}{∥}_{2}{\})}^{2}{]}^{-n_{R}}. \end{array}$$ (19)

We can use this result to obtain an estimate of the spectral bound in (18), writing any code in the block form. It also can be used to derive results for STBCs, defined with block matrices. In the sequence, we will present some examples of how this is done for several well-known codes.

**i**) (*QOSTBC for n*_*T*_ = 4) Consider the Alamouti block

$$\begin{array}{cc} C_{pq} & =(\begin{array}{cc} x_{p} & -x_{q}^{*}\\ x_{q} & x_{p}^{*} \end{array}). \end{array}$$

As we have seen in Example 4 of last section, the following STBC for *n*_*R*_ = *n*_*T*_ = *k* = 4 is due to Jafarkhani [52], with the following code:

$$\begin{array}{cc} C & =(\begin{array}{cc} C_{12} & -C_{34}^{*}\\ C_{34} & C_{12}^{*} \end{array})=(\begin{array}{cccc} x_{1} & -x_{2}^{*} & -x_{3}^{*} & x_{4}\\ x_{2} & x_{1}^{*} & -x_{4}^{*} & -x_{3}\\ x_{3} & -x_{4}^{*} & x_{1}^{*} & -x_{2}\\ x_{4} & x_{3}^{*} & x_{2}^{*} & x_{1} \end{array}). \end{array}$$

Note that C is a rate one Quasi-orthogonal STBC. This means the *ML* decoding may be done for groups of symbols independently. Using properties of the spectral norm, one can find an upper bound for pairwise error probability of C. Let A,E∈C and let Δ = *A* − *E*. We have

$$\begin{array}{cc} {∥\Delta ∥}_{2} & ={∥(\begin{array}{cc} A_{12}-E_{12} & -A_{34}^{*}+E_{34}^{*}\\ A_{34}-E_{34} & A_{12}^{*}-E_{12}^{*} \end{array})∥}_{2}\\ & ={∥(\begin{array}{cc} \Delta_{12} & -\Delta_{34}^{*}\\ \Delta_{34} & \Delta_{12}^{*} \end{array})∥}_{2}\\ & \leq {∥(\begin{array}{cc} \Delta_{12} & 0\\ 0 & \Delta_{12}^{*} \end{array})∥}_{2}+{∥(\begin{array}{cc} 0 & -\Delta_{34}^{*}\\ \Delta_{34} & 0 \end{array})∥}_{2} \end{array}$$

Supposing we have a real constellation (BPSK for instance), it follows that *A* = A*, and then,

$${∥\Delta ∥}_{2}\leq \text{max}\{∥\Delta_{12}{∥}_{2},∥\Delta_{12}{∥}_{2}\}+\text{max}\{∥\Delta_{34}{∥}_{2},∥\Delta_{34}{∥}_{2}\}=∥\Delta_{12}{∥}_{2}+{∥\Delta_{34}∥}_{2}.$$

Thus, using the spectral bound for the PEP (19),

$$\begin{array}{cc} P(X\to E) & \leq (1+\gamma_{d}{\left||\Delta |\right|}_{2}^{2}{)}^{-n_{R}}\\ & =[1+\gamma_{d}(∥\Delta_{12}{∥}_{2}^{2}+2∥\Delta_{12}{∥}_{2}∥\Delta_{34}{∥}_{2}+∥\Delta_{34}{∥}_{2}^{2}{)]}^{-n_{R}}, \end{array}$$

which is lower than *PEP* for the Alamouti code *C*_*pq*_, for example.

**ii**) (*QOSTBC for n*_*T*_ = 8) Another QOSTBC, whose codewords are block matrices, is also given by Jafarkhani [52]. Define the block

$$\begin{array}{cc} C_{abcd} & =(\begin{array}{cccc} x_{a} & -x_{b}^{*} & -x_{c}^{*} & x_{d}\\ x_{b} & x_{a}^{*} & -x_{d}^{*} & -x_{c}\\ x_{c} & -x_{d}^{*} & x_{a}^{*} & -x_{b}\\ x_{d} & x_{c}^{*} & x_{b}^{*} & x_{a} \end{array}). \end{array}$$

The code in [52] is given by

$$\begin{array}{cc} C & =(\begin{array}{cc} C_{1234} & -C_{5678}^{*}\\ C_{5678} & C_{1234}^{*} \end{array}). \end{array}$$

Similarly to what we did in the previous QOSTBC, we can compute the spectral norm of the difference Δ of the codewords *X* ≠ *E* for a real constellation with

$$\begin{array}{cc} {∥\Delta ∥}_{2} & ={∥(\begin{array}{cc} \Delta_{1234} & -\Delta_{5678}^{*}\\ \Delta_{5678} & \Delta_{1234}^{*} \end{array})∥}_{2}\leq {∥(\begin{array}{cc} \Delta_{1234} & 0\\ 0 & \Delta_{1234}^{*} \end{array})∥}_{2}+{∥(\begin{array}{cc} 0 & -\Delta_{5678}^{*}\\ \Delta_{5678} & 0 \end{array})∥}_{2}\\ & =\text{max}\{∥\Delta_{1234}{∥}_{2},∥\Delta_{1234}{∥}_{2}\}+\text{max}\{∥\Delta_{5678}{∥}_{2},∥\Delta_{5678}{∥}_{2}\}\\ & =∥\Delta_{1234}{∥}_{2}+{∥\Delta_{5678}∥}_{2}. \end{array}$$

Each of the terms Δ_*abcd*_ can be calculated using the norm of the codewords from the last example. We obtain

$${∥\Delta ∥}_{2}\leq ∥\Delta_{12}{∥}_{2}+∥\Delta_{34}{∥}_{2}+∥\Delta_{56}{∥}_{2}+{∥\Delta_{78}∥}_{2}$$

Thus, using the spectral bound again for the PEP (19),

$$\begin{array}{c} P\left(X\to E\right)\leq (1+\gamma_{d}{\left||\Delta |\right|}_{2}^{2}{)}^{-n_{R}}\\ ={\left[1+\gamma_{d}(\sum_{i=1}^{3}∥\Delta_{\left(2i+1)(2i+2\right)}{∥}_{2}^{2}+\sum_{\begin{array}{c} i\neq j\\ i,j\leq 3 \end{array}}2∥\Delta_{\left(2i+1)(2i+2\right)}{∥}_{2}{∥\Delta_{\left(2j+1)(2j+2\right)}∥}_{2})\right]}^{-n_{R}} \end{array}$$

which is lower than the *PEP* for the Alamouti code *C*_*pq*_, and the QOSTBC of the Example (i).

**Fig 17:**
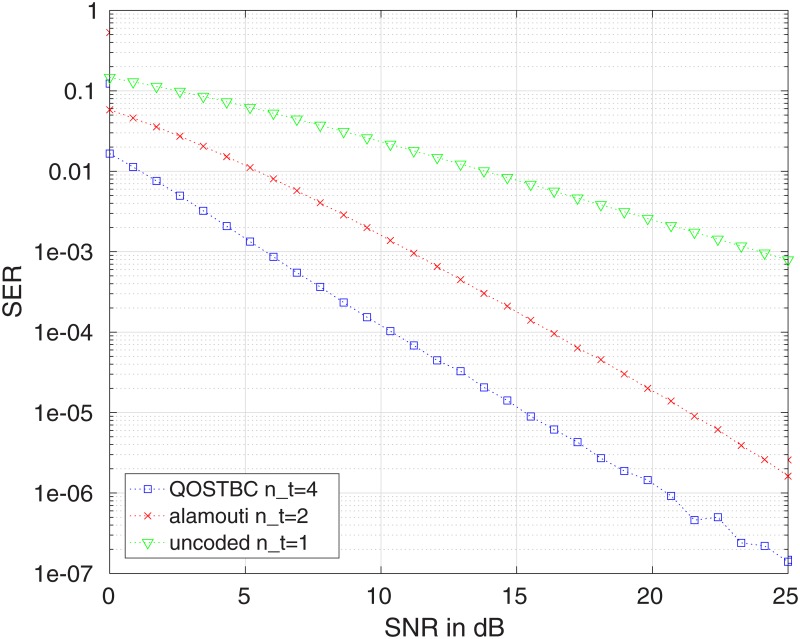
Symbol error probability for the QOSTBC with *n*_*T*_ = 4 compared with the Alamouti code and an uncoded channel. All codes use BPSK modulation, one receive antenna and Rayleigh fading.

**Fig 18:**
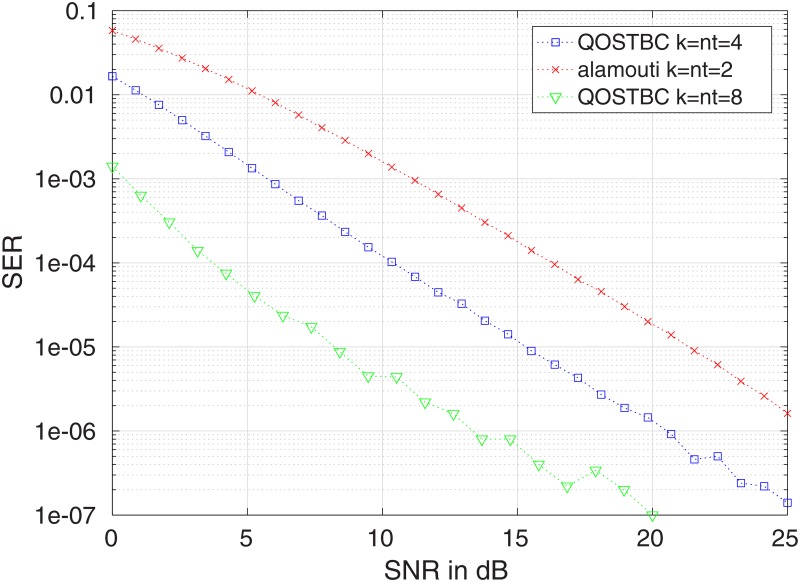
Symbol error probability for the QOSTBC with *n*_*T*_ = 8 compared with the Alamouti code and the 4 × 4 QOSTBC of (i). All codes use BPSK modulation, one receive antenna and Rayleigh fading.

Fig 17 compares the SER for the 4 × 4 QOSTBC presented in (i) and the Alamouti code, showing that the first possesses a lower error probability. In Fig 18, we compare the 8×8 QOSTBC of (ii), with the Alamouti code and the 4 × 4 QOSTBC of (i). In both cases, we assume BPSK modulation and, in both cases, we consider the spectral norm for the comparisons.

## 8 Conclusions

A natural environment where the space-time codes live in is proposed. A new design criterion for space-time block codes for multi-antenna communication systems on coherent Rayleigh fading channels is obtained. This criterion aims at minimizing the pairwise error probability of the maximum likelihood decoder, endowed with the matrix spectral norm. The random matrix theory is used, and a very useful approximation function for the probability density function of the largest eigenvalue of a Wishart Matrix is given, and an approximation for the pairwise error probability function for the spectral case is obtained. The proposed criterion can be used to choose the best STBC in a given family of matrices. The choice is based on the pairwise error probability, serving as a tool to find new codes. Several known and new STBCs were also analyzed in terms of the largest eigenvalue criterion and comparisons were done with the classical criteria.

## References

[1] Shannon C. Mathematical theory of communications. Bell Systems Tech J. 1948;27: 379–423, 623–656. 10.1002/j.1538-7305.1948.tb01338.x

[2] Foschini GJ and Gans MJ. On limits of wireless communications in a fading environment when using multiple antennas. Wirel Pers Commun. 1998;6: 311–335. 10.1023/A:1008889222784

[3] Telatar IE. Capacity of multi-antenna Gaussian channels. European Trans on Telecomm. 1999;10(6): 585–595. 10.1002/ett.4460100604

[4] Tarokh V, Seshadri N and Calderbank AR. Space-time codes for high data rate wireless communication: Performance Criterion and Code Construction. IEEE Trans Inf Theory. 1998;44(2): 744–765. 10.1109/18.661517

[5] Alamouti SM. A simple transmit diversity technique for wireless communications. IEEE J Sel Top Comm. 1998;16(8): 1451–1458. 10.1109/49.730453

[6] Tarokh V, Jafarkani H and Calderbank AR. Space-time block codes from orthogonal designs. IEEE Trans Inf Theory. 1999;45(5): 1456–1467. 10.1109/18.771146

[7] Yuan J, Chen Z and Vucetic B. Performance and design of space-time Coding in fading channels. IEEE Trans Commun. 2003;51(12): 1991–1996. 10.1109/TCOMM.2003.820741

[8] Tirkkonen O and Hottinen A. Square-matrix embeddable space-time block codes for complex signal constellations. IEEE Trans Inf Theory. 2002;48(2): 384–395. 10.1109/18.978740

[9] Wishart J. The generalized product moment distribution in samples from a normal multivariate population. Biometrika 1928;20A: 32–43. 10.1093/biomet/20A.1-2.32

[10] Wigner EP. On the Statistical distribution of the widths and spacings of nuclear resonance levels. Proc Camb Philos Soc. 1951;47: 790–798. 10.1017/S0305004100027237

[11] Wigner EP. Characteristic vectors of bordered matrices with infinite dimensions. Ann Math. 1955;62: 548–564. 10.2307/1970079

[12] Wigner EP. On the distribution of the roots of certain symmetric matrices. Ann Math. 1958;67: 325–327. 10.2307/1970008

[13] Dyson F. A Brownian-motion model for the eigenvalues of a random matrix. J Math Phys. 1962;3: 1191–1198. 10.1063/1.1703862

[14] Dyson F. Statistical theory of the energy levels of complex systems, I-III. J Math Phys. 1962;3: 140–156, 157–165, 166–175. 10.1063/1.1703773

[15] Dyson F. The threefoldway algebraic structure of symmetry groups and ensembles in quantum mechanics. J Math Phys. 1962;3: 1199–1215. 10.1063/1.1703863

[16] Tao T and Vu V. Random Matrices: the distribution of the smallest singular values. Geom Funct Anal. 2010;20: 260–297. 10.1007/s00039-010-0057-8

[17] Tao T and Vu V. The Wigner-Dyson-Mehta bulk universality conjecture for Wigner Matrices. Electron J Probab. 2011;16: 2104–2121. 10.1214/EJP.v16-944

[18] Tao T and Vu V. Random Matrices: Universality of local eigenvalue statistics. Acta Math. 2011;206: 127–204. 10.1007/s11511-011-0061-3

[19] Tao T and Vu V. A Central limit theorem for the determinant of a Wigner matrix. Adv Math. 2012;231(1): 74–101. 10.1016/j.aim.2012.05.006

[20] Forrester PJ. Log-gases and random matrices. Princeton Princeton University Press, 2010.

[21] Tulino AM and Verdú S. Random matrix theory and wireless communications. Foundations and Trends in Communications and Information Theory, 2004 10.1561/0100000001

[22] Hsu PL. On the distribution of the roots of certain determinantal equations. Ann Eugen. 1939;9: 250–258. 10.1111/j.1469-1809.1939.tb02212.x

[23] Jonsson D. Some limit theorems for the eigenvalue of a sample covariance matrix. J Multivar Anal. 1982;12: 1–38. 10.1016/0047-259X(82)90080-X

[24] Marcenko VA and Pastur LA. Distributions of eigenvalues for some sets of random matrices. Math USSR-Sb. 1967;1: 457–483. 10.1070/SM1967v001n04ABEH001994

[25] Trotter HF. Eigenvalue distributions of large hermitian matrices Wigner semi-circle law and theorem of Kac, Murdock and Szego. Adv Math.1984;54: 67–82. 10.1016/0001-8708(84)90037-9

[26] Wachter KW. The strong limits of random matrix spectra for sample matrices of independent elements. Ann Probab. 1978;6: 1–18. 10.1214/aop/1176995607

[27] German S. A limit theorem for the norm of random matrices. Ann Probab. 1980;8: 252–261. 10.1214/aop/1176994775

[28] Sugiyama T. On the distribution of the largest root of the covariance matrix. Ann Math Stat. 1967;38, 1148–1151. 10.1214/aoms/1177698783

[29] Krishnaiah PR and Cheng TC. On the exact distribution of the smallest roots of the Wishart Matrix using zonal polynomials. Ann Inst Stat Math. 1971;23: 293–295. 10.1007/BF02479230

[30] Silverstein JW. The smallest eigenvalue of a large-dimensional Wishart Matrix. Ann Probab. 1985;13: 1364–1368. 10.1214/aop/1176992819

[31] Vlok JD. Analytic approximation to the largest eigenvalue distribution of a white Wishart matrix. IET Comm. 2012;6(12): 1804–1811. 10.1049/iet-com.2011.0843

[32] Chiani M. Distribution of the largest eigenvalue for real Wishart and Gaussian random matrices and a simple approximation for the Tracy-Widom distribution. J Multivar Anal. 2014;129: 69–81. 10.1016/j.jmva.2014.04.002

[33] Alfano G, Lozano A, Tulino AM and S. Verdú. Mutual information and eigenvalue distribution of MIMO rician channels. International Symposium on Information Theory and its Applications, ISITA, 2004.

[34] Chiani M, Win MZ and Zanella A. On the capacity of spatially correlated MIMO Rayleigh fading channels. IEEE Trans Inf Theory. 2003;49(10): 2363–2371. 10.1109/TIT.2003.817437

[35] Ahmadi A. A new approach to fast decode quasi-orthogonal space-time block codes. IEEE Trans Wirel Commun. 2015;14(1): 165–176. 10.1109/TWC.2014.2334615

[36] Raleigh GG and Cioffi JM. Spatio-temporal coding for wireless communication. IEEE Trans Commun. 1998;46(3): 357–366. 10.1109/26.662641

[37] Gesbert D, Kountouris M, Heath RW Jr, Chae C and Chae T. Shifting the MIMO paradigm. IEEE Signal Process Mag. 2007;24(5): 36–46. 10.1109/MSP.2007.904815

[38] Caire G and Shamai S. On the achievable throughput of a multi-antenna gaussian broadcast channel. IEEE Trans Inf Theory. 2003;49(7): 1691–1706. 10.1109/TIT.2003.813523

[39] Viswanath P and Tse DNC. Sum capacity of a vector gaussian broadcast channel and uplink-downlink duality. IEEE Trans Inf Theory. 2003;49(8): 1912–1921. 10.1109/TIT.2003.814483

[40] Vishwanath S, Jindal N and Goldsmith A. Duality, achievable rates, sum-rate capacity of gaussian MIMO broadcast channels. IEEE Trans Inf Theory. 2003;49(10): 658–668. 10.1109/TIT.2003.817421

[41] Larsson EG, Tufvesson F, Edfors O and Marzetta TL. Massive MIMO for next generation wireless systems. IEEE Commun Mag. 2014;52(2): 186–195. 10.1109/MCOM.2014.6736761

[42] Marzetta TL. How much training is required for multiuser MIMO. Proc. 40th Asilomar Conf. Signals, Syst., Comput., Nov. 2006; 359–363.

[43] Marzetta TL. Noncooperative cellular wireless with unlimited numbers of base station antennas. IEEE Trans Wirel Commun. 2010;9(1): 3590–3600. 10.1109/TWC.2010.092810.091092

[44] Rusek F, Persson D, Lau BK, Larsson EG, Marzetta TL, Edfors O, et al Scaling up MIMO: opportunities and challenges with very large arrays. IEEE Signal Process Mag. 2013;30(1): 40–60. 10.1109/MSP.2011.2178495

[45] Björnson E, Hoydis J and Sanguinetti L. Massive MIMO has unlimited capacity. IEEE Trans Wirel Commun. 2018;17(1): 574–590. 10.1109/TWC.2017.2768423

[46] Vucetic B and Yuan J. Space-time coding. Wiley, 2003.

[47] Horn RA and Johnson CR. Matrix analysis. Cambridge University Press, 1990.

[48] Edelman A. Eigenvalues and condition numbers of random matrices. SIAM J Matrix Anal Appl. 1988;9(4): 543–560. 10.1137/0609045

[49] Zanella A and Chiani M. The PDF of the Ith largest eigenvalue of central Wishart matrices and its application to the performance analysis of MIMO channels. GLOBECOM, New Orleans, 2008.

[50] Zanella A, Chiani M and Win MZ. On the marginal distribution of the eigenvalues of Wishart Matrices. IEEE Trans Commun. 2009;57(4): 1050–1060. 10.1109/TCOMM.2009.04.070143

[51] Biglieri E. Coding for wireless channels. Springer, 2005.

[52] Jafarkhani H. A quasi-orthogonal space-time block code. IEEE Trans Commun. 2001;49(1): 1–4. 10.1109/26.898239

[53] Hughes BL. Optimal space-time constellations from groups. IEEE Trans Inf Theory. 2003;49(2): 401–410. 10.1109/TIT.2002.807283

[54] Grover R, Su W and Pados DA. An 8 × 8 quasi-orthogonal STBC form for transmissions over eight or four antennas. IEEE Trans Wirel Commun. 2008;7(12): 4777–4785. 10.1109/T-WC.2008.070791

